# Integration of multiple imaging platforms to uncover cardiovascular defects in adult zebrafish^[Author-notes cvab310-FM2]^

**DOI:** 10.1093/cvr/cvab310

**Published:** 2021-10-05

**Authors:** Anabela Bensimon-Brito, Giulia L M Boezio, João Cardeira-da-Silva, Astrid Wietelmann, Srinath Ramkumar, Pia R Lundegaard, Christian S M Helker, Radhan Ramadass, Janett Piesker, Arno Nauerth, Clemens Mueller, Didier Y R Stainier

**Affiliations:** Department of Developmental Genetics, Max Planck Institute for Heart and Lung Research, Bad Nauheim, Germany; DZHK German Centre for Cardiovascular Research, Partner Site Rhine-Main, Bad Nauheim, Germany; Department of Developmental Genetics, Max Planck Institute for Heart and Lung Research, Bad Nauheim, Germany; DZHK German Centre for Cardiovascular Research, Partner Site Rhine-Main, Bad Nauheim, Germany; Department of Developmental Genetics, Max Planck Institute for Heart and Lung Research, Bad Nauheim, Germany; DZHK German Centre for Cardiovascular Research, Partner Site Rhine-Main, Bad Nauheim, Germany; Scientific Service Group MRI and µ-CT, Max Planck Institute for Heart and Lung Research, Bad Nauheim, Germany; Department of Developmental Genetics, Max Planck Institute for Heart and Lung Research, Bad Nauheim, Germany; DZHK German Centre for Cardiovascular Research, Partner Site Rhine-Main, Bad Nauheim, Germany; Department of Life Sciences, Goethe University Frankfurt am Main, Frankfurt, Germany; Laboratory for Molecular Cardiology, Department of Cardiology, Vascular, Pulmonary and Infectious Diseases, University Hospital of Copenhagen, Copenhagen, Denmark; Department of Biomedical Sciences, University of Copenhagen, Copenhagen, Denmark; Department of Developmental Genetics, Max Planck Institute for Heart and Lung Research, Bad Nauheim, Germany; Department of Developmental Genetics, Max Planck Institute for Heart and Lung Research, Bad Nauheim, Germany; Scientific Service Group Microscopy, Max Planck Institute for Heart and Lung Research, Bad Nauheim, Germany; Bruker Biospin MRI GmbH, Ettlingen, Germany; Department of Radiology, Kerckhoff-Klinik, Bad Nauheim, Germany; Department of Developmental Genetics, Max Planck Institute for Heart and Lung Research, Bad Nauheim, Germany; DZHK German Centre for Cardiovascular Research, Partner Site Rhine-Main, Bad Nauheim, Germany

**Keywords:** Cardiovascular, Pre-clinical, Multi-modal imaging, Electrophysiology, MRI, TGFß signalling, Aorta

## Abstract

**Aims:**

Mammalian models have been instrumental in investigating adult heart function and human disease. However, electrophysiological differences with human hearts and high costs motivate the need for non-mammalian models. The zebrafish is a well-established genetic model to study cardiovascular development and function; however, analysis of cardiovascular phenotypes in adult specimens is particularly challenging as they are opaque.

**Methods and results:**

Here, we optimized and combined multiple imaging techniques including echocardiography, magnetic resonance imaging, and micro-computed tomography to identify and analyse cardiovascular phenotypes in adult zebrafish. Using *alk5a/tgfbr1a* mutants as a case study, we observed morphological and functional cardiovascular defects that were undetected with conventional approaches. Correlation analysis of multiple parameters revealed an association between haemodynamic defects and structural alterations of the heart, as observed clinically.

**Conclusion:**

We report a new, comprehensive, and sensitive platform to identify otherwise indiscernible cardiovascular phenotypes in adult zebrafish.

## Graphical Abstract

**Graphical Abstract:**
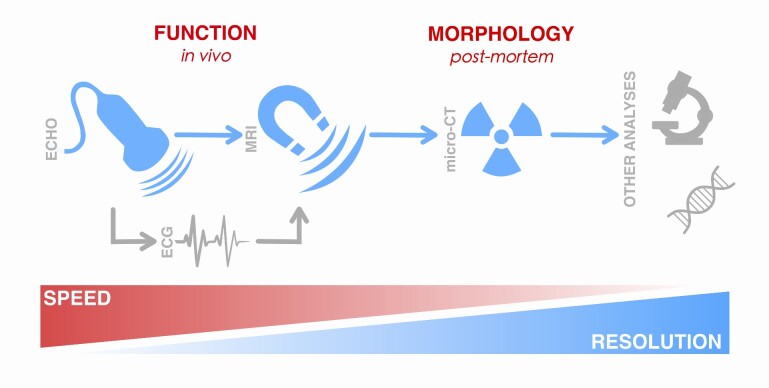

## 1. Introduction

Cardiovascular diseases are a major cause of death worldwide.^1^ The understanding of their pathophysiology has relied on the identification and analysis of genes triggering the initiation and progression of cardiovascular defects. Several mammalian models including mice, rats, rabbits, pigs, sheep, and dogs, have been used to investigate various cardiovascular diseases, each with its own benefits and shortcomings. For instance, rodent and human hearts differ in their electrophysiology and oxygen consumption,^2,3^ while large animals have considerable limitations in terms of cost, handling difficulties, and genetic manipulation.^3,4^ Therefore, there is a need for additional animal models to investigate the mechanisms underlying cardiovascular diseases.

The zebrafish is a popular genetic model to study cardiovascular development, disease, and regeneration.^5–8^ External fertilization and optical transparency during embryogenesis allow for *in vivo* analysis of cardiovascular development at single-cell resolution.^9–11^ Previous studies have also highlighted the similarities between the mature human and zebrafish hearts, including comparable beating rates and action potential profiles.^12–19^ Importantly, mutating zebrafish orthologues of human disease genes can accurately model cardiovascular disorders, as observed with mutations leading to arrhythmogenic, dilated and Titin-associated cardiomyopathies.^20–25^ Furthermore, unlike their mammalian counterparts, adult zebrafish hearts can regenerate, thereby providing a unique platform to understand mechanisms of cardiac regeneration.^26–28^ Together, these studies indicate that adult zebrafish have the unique potential to provide high-throughput, translatable scientific knowledge for pre-clinical and clinical cardiovascular research.^21,29,30^ However, morphological and functional characterization of adult zebrafish is particularly challenging as they are no longer optically transparent and too small for most conventional pre-clinical imaging platforms.^31–35^ In addition, many cardiovascular disorders are associated with inherited genetic mutations,^36^ often leading to variable phenotypic expressivity.^37^ Therefore, to understand the cardiovascular phenotypes despite this variability between individuals, it is essential to extract all of the relevant information from a relatively large sample size, which is not easily done with adult specimens.

Due to the aforementioned imaging limitations, most studies of adult cardiac phenotypes in zebrafish rely on histological analyses of tissue sections, which prevent three-dimensional (3D) representation of the morphological features or assessment of cardiac function. In a clinical setting, diagnosing the onset and progression of cardiovascular diseases often requires a combination of multiple imaging analyses including echocardiography, magnetic resonance imaging (MRI), and computed tomography (CT).^38–41^ Recently, researchers have adapted light-sheet microscopy,^42^ MRI,^32,33,43,44^ and micro-computed tomography (µ-CT)^30,45,46^ to define the morphology of zebrafish organs. Others have used echocardiography in adult zebrafish to characterize the functional impact of cardiac disease or regeneration following injury.^31,47–57^ However, a systematic workflow to study cardiac and cardiovascular phenotypes in adult zebrafish has yet to emerge.

Here, we established new imaging protocols and combined functional analyses of the adult zebrafish heart using echocardiography and *in vivo* MRI with a morphological characterization of the cardiac compartments and adjacent vessels using µ-CT. As a proof-of-concept, we examined *alk5a/tgfbr1a*^–/–^ zebrafish as *TGFBR1* variants in humans have been associated with aortic aneurysms and diseases of the great vessels, the diagnosis of which is most often recognized in young adults.^58–62^ We show that the use of this multimodal preclinical imaging strategy allows for a robust dissection of adult zebrafish cardiovascular phenotypes, even in the presence of high variability between samples with the same genotype at the disease locus.

## 2. Methods

### 2.1 Zebrafish husbandry and lines

All zebrafish husbandry was performed under standard conditions, and all experiments were conducted in accordance with institutional (MPG) and national ethical and animal welfare guidelines (Proposal number B2/1055). All procedures conform to the guidelines from Directive 2010/63/EU of the European Parliament on the protection of animals used for scientific purposes.

All the zebrafish used in the study were in the *Tg(kdrl: GFP)^s843^*^63^ background. The *alk5a* mutant (*tgfbr1a ^bns329^*) zebrafish^64^ carry a 2 bp insertion and a 6 bp deletion in exon 1. Unless otherwise noted, maternal zygotic *alk5a*^*–/–*^ zebrafish were analysed and compared to WT zebrafish. When required, the mutants were genotyped as previously described, using high-resolution melt analysis (HRMA) (Eco-Illumina) and/or sequencing with the following primers: *alk5a* HRM fw: 5′-CTTCTGGACAGACCGTGACA-3′, *alk5a* HRM rv: 5′-GAAGGAGCGCACTGGAAAG-3′.

### 2.2 Confocal microscopy for live imaging of zebrafish larvae

For live confocal imaging, embryos and larvae were embedded in 1% low-melting agarose/egg water with 0.2% tricaine (Sigma) to image stopped hearts. Larvae were imaged with a Zeiss LSM800 Axio Examiner confocal microscope with a W Plan-Apochromat 40×/1.0 or W Plan-Apochromat 20×/1.0 dipping lens. In order to image the heart, the larvae were placed in a supine position. All images were acquired using the ZEN Blue (Zeiss) software.

### 2.3 Histology and immunostaining

Adult hearts were fixed in 4% buffered paraformaldehyde for 1 h at room temperature, washed in 1× PBS and embedded as previously described.^65^ Briefly, the tissue was placed overnight at 4°C in a solution of 30% (w/v) sucrose prepared in 1× PBS, pre-embedded in 7.5% (w/v) porcine gelatin (Sigma)/15% (w/v) sucrose in 1× PBS at 37°C for 1 h and embedded with a new solution of gelatin. Tissue blocks were frozen in isopentane (Sigma) cooled in liquid nitrogen. Cryosections were cut at 10 µm using a Leica CM3050S cryostat (Leica) and kept at −20°C until further use. Prior to processing, the slides were thawed for 10 minutes at room temperature and the gelatin was removed in 1× PBS at 37°C.

For haematoxylin and eosin staining, the cryosections were stained with acidic hemalum (Waldeck) for 10 min, washed in running tap water for 2 min and rinsed in deionized water. The sections were then counterstained with eosin (Waldeck) for 6 min, dehydrated in 100% ethanol, cleared in xylene and mounted in entellan (Merck). The sections were imaged using a Nikon SMZ25 microscope.

Immunostaining started with a wash in 0.1M glycine (Sigma) followed by permeabilization for 7 minutes at –20°C in pre-cooled acetone. The sections were incubated in a blocking solution of PBDX (1% (w/v) Bovine Serum Albumin, 1% (v/v) DMSO, 1% (v/v) Triton-X100 in PBS) with 15% (v/v) goat serum for a minimum of two hours at room temperature. Incubation with the following primary antibody was performed overnight at 4°C: anti-GFP chicken (Aves Technology; 1:400), anti-Elastin2 rabbit^66^ (1:100). The slides were washed several times with PBDX and incubated with the corresponding secondary antibodies (1:500) overnight at 4°C: anti-chicken AlexaFluor 488 (ThermoFisher), anti-rabbit AlexaFluor 647 (ThermoFisher). For all incubations, the slides were covered with a piece of Parafilm-M to ensure homogenous distribution of the solution. The slides were washed a minimum of 3 times for 15 minutes each in a solution of 0.3% (v/v) Triton-X100 in PBS (PBST) and counterstained with 0.0002% (w/v) DAPI (Merck) in PBST for 10 minutes. The slides were then washed a minimum of 3 times for 15 minutes each in PBST and mounted with DAKO Fluorescence mounting medium (Agilent). Elastin2 antibody was purified from the previously described serum stock.^66^ The sections were imaged using a Zeiss LSM700 microscope, and the ZEN software (Black edition, Zeiss).

### 2.4 Transmission Electron Microscopy (TEM)

Adult hearts were dissected and fixed in 4% PFA with 2.5% glutaraldehyde in 0.05 M HEPES buffer (pH 7.2) for 2 hours at room temperature, and subsequently stored at 4°C. The samples were rinsed three times in 0.05 M HEPES buffer (pH 7.2) and post-fixed in 1% (w/v) OsO_4_ for 1 hour. After washing three times with distilled water, the blocks were stained with 2% uranyl acetate for 1 hour. The samples were dehydrated through a graded series of ethanol washes, transferred to propylene oxide, and embedded in Epon according to standard procedures.^67^ Tissue semi-thin sections (900 nm thick) were obtained in a Ultracut E microtome (Reichert-Jung, Leica) and stained with Richardson staining solution.^68^ Ultra-thin 70 nm sections were then collected on copper grids. After post-staining with uranyl acetate and lead citrate, the ultra-thin sections were examined with a JEM-1400 Plus transmission electron microscope (Jeol, Japan), operated at an accelerating voltage of 120 kV. Digital images were recorded with an EM-14800Ruby Digital CCD camera unit.

### 2.5 Body volume measurements

Total body volume was determined by submerging each animal in a 2 mL syringe filled with water and determining water displacement (1 mL water displacement corresponds to 1 cm^3^ body volume).

### 2.6 Doppler echocardiography

Zebrafish were anaesthetized in system water with 0.016% buffered Tricaine. Zebrafish were placed in a supine position in a bed made of modelling clay, adjustable to the size of the zebrafish, and submerged in anaesthesia solution to ensure the propagation of the ultrasound signal. A Vevo2100^®^ Imaging System (VisualSonics) and the VisualSonics Ultrasound Imaging Software (Version 1.6.0) were used for echocardiography. Since the required ultrasound frequency depends on the size of the sample,^54^ we used a high-frequency MicroScan transducer (MS700 v3.0) at a frequency of 40 MHz, as opposed to the 2–15 MHz used for humans. The transducer was oriented along the longitudinal axis of the zebrafish and the images were acquired with the anterior side of the zebrafish to the left (Supplementary material online, *Figure S2A*). Blood flow imaging and quantification of the haemodynamic parameters were performed in Pulsed-wave (PW) Colour Doppler mode. For optimal quality, imaging was acquired with a field of view of 6.00 × 6.73 mm, Doppler gain of 34 dB and 2D Gain of 48 dB, half line density and low persistence. Videos were recorded for 10 seconds and across different 2D planes, spanning the atrium, ventricle and OFT. At least two videos per area were acquired and used to determine an average of all measurements and increase reproducibility. Image acquisition was completed within 5 minutes of sedation to avoid cardiac function aberrations. After imaging, the zebrafish were returned to system water and observed until recovery. All zebrafish recovered after imaging. To minimize variability between individuals, all conditions including anaesthesia concentration and temperature were maintained constant throughout the measurements. The Vevo Lab™ software package v.1.7.0 (VisualSonics) was used for image analysis.

The heart rate was calculated as beats per minute in the 10 s time frame recorded. The inflow and outflow areas were measured in the 2D planes exhibiting the highest blood velocity across the heart. The regurgitation fractions across the AVC and OFT were calculated as the ratios between the area of the inflow and outflow present simultaneously in the same plane spanning the AV or OFT canal, respectively. The area of the aortic flow refers to the area of flow rostral to OFT, if detected. Values of velocity time integral (VTI), mean and peak gradient, mean and peak velocity, and ejection time were calculated automatically using the Vevo Lab™ software package v.1.7.0 (VisualSonics) and refer to the curves highlighted in Supplementary material online, *Figure S2C,C’*.

#### 2.6.1 Sequential echocardiography measurements and isoprenaline treatments

In order to follow the progression of the phenotype over time, WT and *alk5a*^–/–^ zebrafish were analysed with pulsed-wave colour Doppler echocardiography (as described above), at time 1 (t1), 3 weeks after t1 (t2), and 2 months after t1 (t3). Prior to each echocardiography session, two groups of zebrafish (7 WT and 7 *alk5a*^*–/–*^) were treated with isoprenaline (+ISO, Sigma, Cat. # I5627), a β-adrenergic agonist analogue of adrenaline, in order to increase heart rate and potentially aggravate the cardiovascular phenotype.^69–72^ Isoprenaline powder was dissolved in water to create a stock solution (10 mM) and kept in the dark until use (within 6 hours). Zebrafish were immersed in system water containing 5 μM isoprenaline (diluted from stock) for 30 minutes prior to echocardiography. The zebrafish were subsequently returned to system water without isoprenaline to recover from the anaesthesia and kept in standard conditions until follow up.

### 2.7 Magnetic Resonance Imaging (MRI)

Cardiac *in vivo* MRI measurements were performed on a 7.0 T Bruker PharmaScan (Bruker Biospin, Ettlingen, Germany) small animal MRI system. The instrument was equipped with a 780 mT/m gradient system, a cryogenically cooled 4 channel phased array element ^1^H receiver-coil (CryoProbe^TM^, Bruker Biospin) and a 72 mm room temperature volume resonator for transmission (Bruker Biospin). Zebrafish were anaesthetized in water with 0.016% buffered Tricaine (Sigma) and placed in a supine position in a bed made of modelling clay. Due to reduced imaging quality using a container with water flow, we decided to analyse our specimens without water flow and reduce the imaging time to less than 20 minutes to ensure animal survival.

Localizer images were acquired using a spin-echo sequence and correction of slice orientation was performed when necessary. In addition, RARE (Rapid Acquisition with Relaxation Enhancement) sequences (TR = 2500 ms, TE = 36.7 ms, slice thickness = 0.3 mm) in a sagittal orientation were used to determine the correct coronal plane for imaging of the ventricle or OFT (Supplementary material online, *Figure S3A–B’*). The imaging was performed with the following parameters: IntraGate 2D FLASH: TE/TR: 4.2/8.2 ms, flip angle 10°, slice thickness: 0.3 mm, in-plane resolution: 59 × 59 or 47 × 47 µm^2^. Navigator based self-gating^73,74^ was used to determine the cardiac rate and to perform cine retrospective reconstruction. Similar to the values observed with echocardiography, heart rates used for reconstruction had a median of 122 and 125 bpm, and minimum and maximum values of 62–172 and 72–200 bpm for the WT and *alk5a*^–/–^ zebrafish, respectively. With the help of the profile images and the navigator signal, we were also able to detect and exclude sudden movements of the anaesthetized zebrafish as well as shifts in resting position (Supplementary material online, *Figure S3 C–C”*).

The evaluation of the functional heart data of adult zebrafish, following MRI, was performed with the Qmass^®^ MR 8.1 (Medis Medical Imaging Systems) software. The percentage of OFT and ventricular expansion was calculated by the software after manually defining the lumen (or wall) outline during maximum and minimum chamber dilation. The OFT and ventricular measurements were performed in different recordings with different orientations selected to show the cross-section of the desired chamber. All the values represent the average of two independent analyses of the same data performed by two different scientists. Inter-observer variability in parameter quantification is presented in *Tables 1 and 2*. Despite the variability between inter-observer measurements, both observers obtained similar sample mean values.

**Table 1:** Inter-observer measurements for ventricular outer wall and luminal expansion

	Ventricular outer wall (%)			Ventricular lumen (%)		
Fish	Sample mean	Observer 1	Observer 2	Sample mean	Observer 1	Observer 2
WT 1		12	8		24	29
WT 2		20	24		56	62
WT 3		1	4		16	26
WT 4		6	1		10	6
WT 5	9.8	3	2	27.4	10	13
WT 6		5	11		22	37
WT 7		5	1		17	16
WT 8		12	12		36	29
WT 9		25	12		31	51
WT 10		20	12		33	23
Mean		10.9	8.7		25.5	29.2

MUT 1		19	19		64	62
MUT 2		4	1		40	35
MUT 3		1	1		53	41
MUT 4		11	11		17	25
MUT 5		6	5		35	28
MUT 6	9.1	12	9	31.3	14	21
MUT 7		7	2		18	23
MUT 8		3	5		34	25
MUT 9		4	8		16	25
MUT 10		40	1		51	21
MUT 11		20	15		19	29
MUT 12		10	4		31	23
Mean		11.4	6.8		32.7	29.8

**Table 2:** Inter-observer measurements for outflow tract outer wall and luminal expansion

	Outflow outer wall (%)			Outflow lumen (%)		
Fish	Sample mean	Observer 1	Observer 2	Sample mean	Observer 1	Observer 2
WT 1		5	3		14	7
WT 2		4	8		12	9
WT 3		3	8		11	6
WT 4		7	6		11	8
WT 5	6.4	11	4	11.5	12	15
WT 6		14	3		13	13
WT 7		5	3		21	15
WT 8		12	8		7	7
WT 9		10	5		14	8
WT 10		3	5		12	14
Mean		7.4	5.3		12.7	10.2

MUT 1		2	4		34	27
MUT 2		11	9		36	27
MUT 3		7	6		41	41
MUT 4		14	10		9	15
MUT 5		5	12		33	35
MUT 6	6.8	8	6	34.1	30	45
MUT 7		2	6		38	40
MUT 8		7	5		34	33
MUT 9		5	3		31	38
MUT 10		9	2		46	30
MUT 11		7	9		44	36
MUT 12		6	9		44	31
Mean		6.9	6.8		35.0	33.2

### 2.8 Micro-CT

After the MRI recordings, the zebrafish were euthanized with an overdose of anaesthetic in water (0.16% Tricaine in system water, buffered to pH 7.0–7.5), fixed in ice-cold 4% PFA and kept in PFA over-night at 4°C. As previously described,^46^ a solution of 2.5% phosphomolybdic acid (PMA) prepared in demineralized water was used to stain each zebrafish individually for a period of 6 days. Samples were then gradually transferred to a 70% ethanol solution where they were preserved at room temperature until imaging.

All samples were scanned using a µ-CT scanner model Skyscan 1276 (Bruker). X-ray parameters for the imaging were: Source voltage = 55 kV; source current = 72 µA; image pixel size = 2.965 µm; filter = Al 0.25 mm; rotation step = 0.25° to 180°. For image reconstruction, we used the NRecon (version 1.7.1.0) and InstaRecon (version 2.0.3.7) softwares. Resulting reconstructed images were 3856 × 2248 pixels, with a pixel size of 2.965 µm.

All the morphological measurements were performed with the Imaris (Bitplane) software. The atrium, ventricle, OFT, and aorta were manually segmented across the entire 3D volume. The lumen of the OFT was also separately segmented. The volumes of each compartment were calculated with the ‘Surface tool’ in Imaris and, when required, normalized to the heart volume (Supplementary material online, *Figure S4C–E*) or total OFT volume (*Figure 4H*). The OFT wall volume was calculated by subtracting the OFT luminal volume from the total OFT volume. The diameter of the aorta was measured in the most proximal third of the aorta.

### 2.9 Electrocardiography (ECG)

Adult zebrafish were anaesthetized in 0.016% of Tricaine for 5 min, and placed dorsal side up in a wet sponge. Anaesthesia was maintained during the recording through oral perfusion with zebrafish tank water containing 0.016% Tricaine using a roller pump (ISM827B, ISMATEC Germany) at a 2 mL/min speed. Two custom-made stainless-steel electrodes were used to record the ECG. One electrode was placed above the cardiac region using micromanipulators (Marzhauser MM33, Marzhauser, Germany). The second electrode was placed as a reference electrode in the sponge submerged in recording solution. The electrodes were connected to a differential AC amplifier (A-M Systems, WA, USA) with the following filter settings: Low cut-off filter =10 Hz; high cut-off filter = 1 KHz; Gain = ×100. Signals were digitized in a PowerLab 4/30 (AD instruments, USA) and recorded at 10 k/s in LabChart 7.0 (AD instruments, USA) using a digital low pass filter (cut-off frequency = 50 Hz; active input amplifier = 5 V range; low pass filter = 200 Hz; Mains filter = active). The zebrafish were recorded for minimum of 5 minutes, and the ECG was analysed in 30 consecutive beats using LabChart 8 (ADInstruments, version 8).

### 2.10 Data analyses and statistics

All statistical analyses were performed in GraphPad Prism (Version 6.07) and illustrations were done in Inkscape (XQuartz X11).

For comparison of WT vs *alk5a*^*–/–*^ samples, a Gaussian distribution was tested for every sample group using the D’Agostino-Pearson omnibus normality test. When both samples passed the normality test, *P*-values were determined by unpaired *t-*test. When at least one of the samples did not pass the normality test, *P*-values were determined using the Mann–Whitney test for comparison of two samples.

To address the linear relationship between all possible pairs of parameters, correlation matrices were generated through the calculation of Pearson’s correlation coefficients. A binary numerical system was used to compute the sex of individuals (0, male; 1, female). Calculations and corresponding correlograms were generated using RStudio v1.1.456 (RStudio Team, 2015), as well as the *Hmisc* (v 4.4–0; Harrel Jr, 2020) and *corrplot* (v0.84; Wei, 2017) packages. The significance level was set to α = 0.05 for all bivariate correlations.

## 3. Results

### 3.1 Selecting a mutant to test the sensitivity of the imaging platform

**Figure 1:**
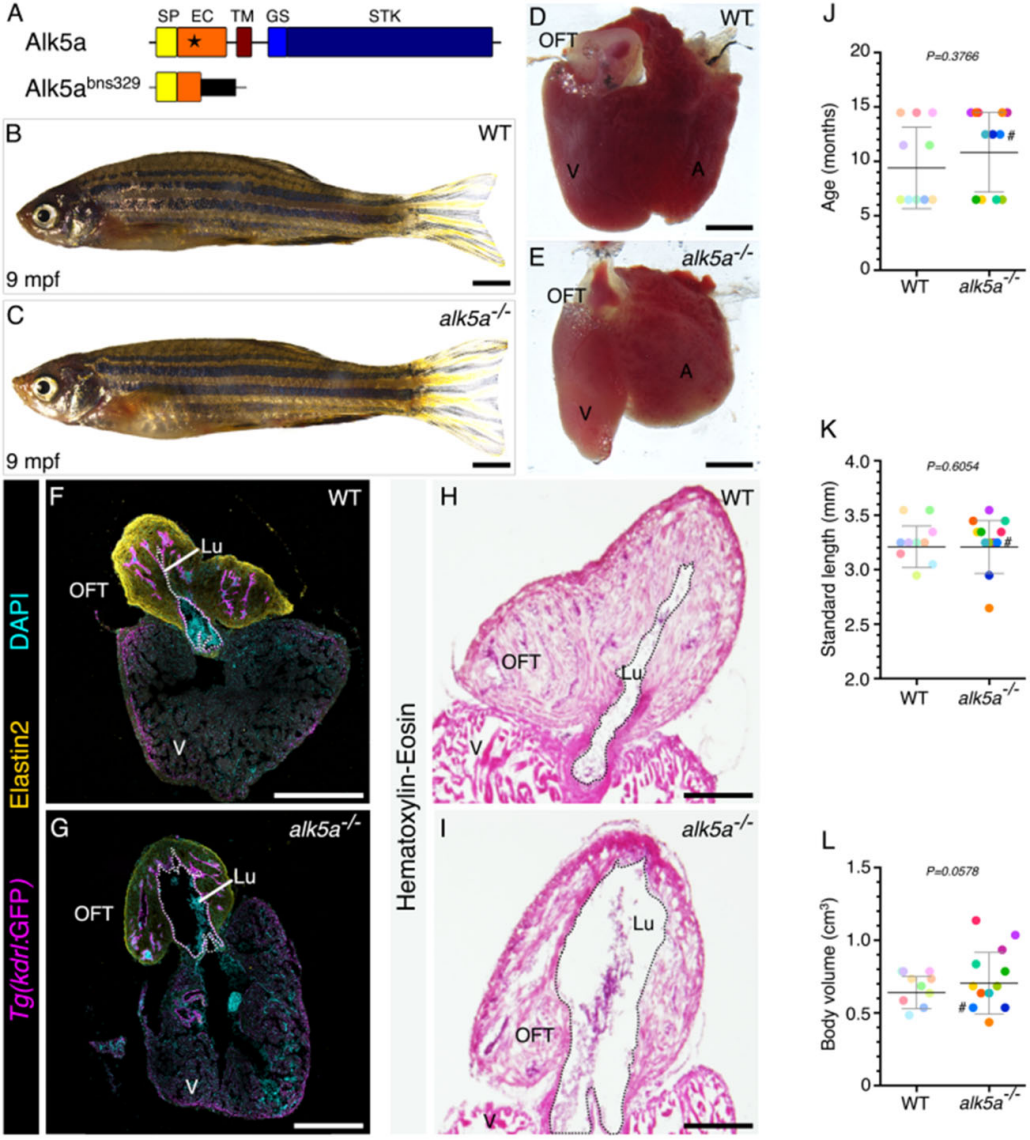
*alk5a* ^–/–^ adult zebrafish display variable cardiac phenotypes without gross morphological defects. (*A*) Schematic of Alk5a WT and mutant proteins depicting each domain and the site of the mutation (star). (*B, C*) Brightfield images of 9 mpf WT (*B*) and *alk5a^–/–^* (*C*) zebrafish. (*D, E*) Brightfield images of *alk5a^–/–^* hearts (*E*) which occasionally exhibit a dilated outflow tract (OFT) lumen compared to WT (D). (F–I) Cryosections of WT (*F, H*) and *alk5a^–/–^* (*G, I*) hearts immunostained for *Tg(kdrl:* eGFP) expression (endothelial cells) and Elastin2 (F, G), and stained for haematoxylin-eosin (*H, I*) showing the expanded OFT lumen (Lu, dashed line) in *alk5a^–/–^* zebrafish. (J–L) Quantification of age (*J*), standard length (*K*), and body volume (*L*) of WT (*n* = 10) and *alk5a^–/–^* (*n* = 12) zebrafish used in the subsequent analyses. Plots show the values for each individual and the mean ± SD; *P*-values were determined by unpaired *t*-test (*J, L*) or Mann–Whitney test (*K*). The colour of each dot refers to the same zebrafish across all graphs. The dot adjacent to the number symbol (#) identifies the individual zebrafish mentioned in the text. Scale bars: 2 mm (*B, C*), 200 µm (*D, E, H, I*), 400 µm (*F, G*). A, atrium; EC, extracellular; GS, glycine–serine rich; SP, signal peptide; STK, serine–threonine kinase; TM, transmembrane; V, ventricle.

In order to test the power of multiple imaging techniques to detect subtle adult phenotypes, we first needed to select a suitable model. Many homozygous mutant zebrafish exhibiting cardiovascular defects at early developmental stages have been reported,^75–79^ and some animals heterozygous for mutations display subtle defects in adult stages.^25,80^ Other zebrafish mutants do not display embryonic phenotypes but exhibit pericardial oedema at adult stages (*Figure 1B,C*),^81^ indicating cardiac function defects. We elected to investigate *alk5a* mutants (*Figure 1A*), a third type of mutant, i.e. one that does not exhibit embryonic phenotypes or pericardial oedema at adult stages but can potentially develop cardiac phenotypes. *alk5a*; *alk5b* double mutants exhibit severe pericardial oedema at early developmental stages, due to a defective outflow tract (OFT).^64^ In contrast, *alk5a* single mutant larvae do not exhibit any obvious defects (Supplementary material online, *Figure S1A,B*), including in the cardiovascular system (Supplementary material online, *Figure S1 C–D’*), and they survive to adulthood. Similarly, *alk5a*^*–/–*^ zebrafish at 9 months post-fertilization (mpf) display no gross morphological defects or pericardial oedema (*Figure 1B,C*). However, analysis of dissected hearts including histological sections suggested that 3/6 *alk5a*^*–/–*^ adult zebrafish have an expanded OFT lumen when compared with wild type (WT; *Figure 1D–I*). The amount of Elastin2, a major component of the ECM in the cardiac OFT, appears to be slightly reduced in the *alk5a*^*–/–*^ OFT wall (*Figure 1F and G*). However, as histological sections do not yield a 3D representation of morphological features, and the size of the OFT lumen depends on the plane of the section, it was not possible to conclude whether the OFT lumen was indeed dilated in *alk5a*^–/–^ zebrafish. Moreover, due to the opacity of adult zebrafish, one cannot use live microscopy to investigate the functional consequences of morphological defects in the cardiovascular system. Therefore, we aimed to adapt and optimize cardiovascular preclinical imaging techniques to collect multiple cardiac parameters (*Table 3*) and analyse adult zebrafish phenotypes. We randomly selected 10 WT and 12 *alk5a*^–/–^ zebrafish, ranging from 6 to 15 mpf (*Figure 1J*, *Table 4*), and tracked each individual throughout all the analyses. To account for any body size differences between the two groups, we measured standard length (Figure*1K*) and body volume (*Figure 1L*), and observed no significant differences between WT and mutant groups.

**Table 3:** Description and abbreviation terms for each parameter

Parameter	Abbreviation	Description
Standard length	Std. length	Length from the anterior tip of the snout to the anterior part of the caudal fin
Body volume	Body vol.	Three-dimensional space occupied by the fish body
Outflow tract velocity time integral	OFT VTI	Area within the Doppler curve (integral). It indicates how far blood travels during the flow period and it is a measure of cardiac systolic function and cardiac output.
Outflow tract mean velocity	OFT mean velocity	Average of the instantaneous velocity from the outflow curve for each heartbeat, measured from all cardiac cycles across 10 s
Outflow tract mean gradient	OFT mean gradient	Average of the instantaneous pressure gradients over the entire outflow Doppler curve, measured from all cardiac cycles across 10 s
Outflow tract velocity peak velocity	OFT peak velocity	Maximum velocity of the blood passing from the ventricle to the outflow tract; V_max_
Outflow tract velocity peak gradient	OFT peak gradient	Pressure gradient calculated from the peak velocity with the Bernoulli equation: 4(V_max_)^2^
Ejection time	Ejection time	Period of blood flow from ventricle to the outflow tract
Heart rate	Heart rate	The speed at which the heart beats measured in beats per minute
Area aortic flow	Area aortic flow	Amount of blood (in a 2D plane) detected in the aorta
Area inflow	Area inflow	Amount of blood (in a 2D plane) going from the atrium to the ventricle
Area outflow	Area outflow	Amount of blood (in a 2D plane) going from the ventricle to the outflow tract
Regurgitation fraction in the atrioventricular canal	Regurg. fraction AVC	Relative amount of blood (in a 2D plane) that returns to the atrium during ventricular contraction (systole); inflow area/retrograde flow area
Regurgitation fraction in the outflow tract	Regurg. fraction OFT	Relative amount of blood (in a 2D plane) that returns to the ventricle after ventricular contraction (systole); outflow area/retrograde flow area
Percentage ventricular luminal expansion	% Vent. lum. exp.	Lumen displacement between minimum and maximum ventricular contraction in a cardiac cycle
Percentage ventricular outer wall expansion	% Vent. outer wall exp.	Outer wall displacement between minimum and maximum ventricular contraction in a cardiac cycle
Percentage outflow tract luminal expansion	% OFT lum. exp.	Lumen displacement between minimum and maximum outflow tract expansion in a cardiac cycle
Percentage outflow tract outer wall expansion	% OFT outer wall exp.	Outer wall displacement between minimum and maximum outflow tract expansion in a cardiac cycle
Heart volume	Heart vol.	Three-dimensional space occupied by the heart—calculated by the sum of the volumes of each cardiac compartment
Ventricular volume	Vent. vol.	Three-dimensional space occupied by the ventricle—including the lumen
Percentage ventricular volume	% Vent. vol.	Relative volume of the ventricle in relation to the entire heart
Atrial volume	Atrial vol.	Three-dimensional space occupied by the atrium—including the lumen
Percentage atrial volume	% Atrial vol.	Relative volume of the atrium in relation to the entire heart
Outflow tract volume	OFT vol.	Three-dimensional space occupied by the outflow tract—including the lumen
Percentage of outflow tract volume	% OFT vol.	Relative volume of the outflow tract in relation to the entire heart
Outflow tract luminal volume	OFT lum. vol.	Three-dimensional space occupied by the outflow tract lumen
Percentage outflow tract luminal volume	% OFT lum. vol.	Relative volume of the outflow tract lumen in relation to the entire outflow tract
Outflow tract wall volume	OFT wall vol.	Three-dimensional space occupied by the outflow tract wall; OFT volume—OFT lumen volume
Aortic diameter	Aortic diam.	Maximum diameter of the aorta measured in the most proximal linear portion of the aorta
Aortic volume	Aortic vol.	Three-dimensional space occupied by the aorta anteriorly to the heart

**Table 4:** Biological features

	Wild type (WT)				*alk5a* ^–/–^				
Parameters	Median	Minimum	Maximum	Inter-quartile range (IQR)	Median	Minimum	Maximum	Inter-quartile range (IQR)	*P*-value
Age (months)	8.5	6.0	14.0	6.0–13.3	12	6.0	14.0	6.0–14.0	0.3766
Std. length (mm)	3.2	2.9	3.5	3.1–3.3	3.3	2.6	3.5	3.2–3.3	0.6054
Body vol. (mm^3^)	0.68	0.45	0.75	0.56–0.74	0.65	0.40	1.10	0.56–0.83	0.0578

### 3.2 Echocardiography reveals variable haemodynamic defects in *alk5a*^*–/–*^ adult zebrafish

**Figure 2:**
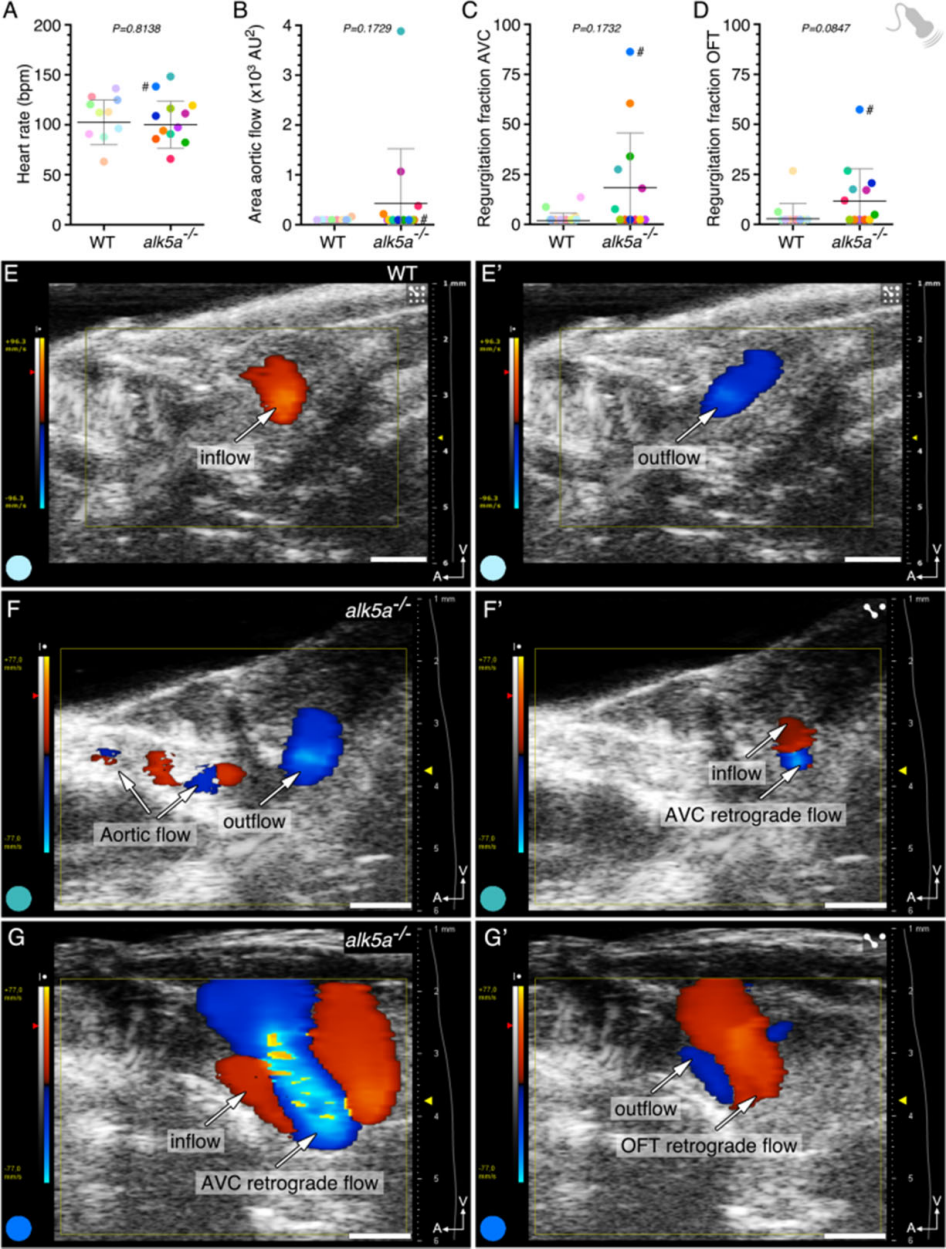
*alk5a* ^–/–^ adult zebrafish display variable haemodynamic defects. (*A–D*) Parameter quantification obtained with echocardiography analyses of WT (*n* = 10) and *alk5a^–/–^* (*n* = 12) adult zebrafish, including heart rate (*A*), area of the aortic flow (*B*), and regurgitation fraction in the AV (*C*) and OFT (*D*) canals. Plots show the values for each individual and the mean ± SD; *P*-values were determined by unpaired *t-*test (*A*) or Mann–Whitney test (*B–D*). (*E, E’*) WT zebrafish exhibit unidirectional blood inflow (red, *E*) and outflow (blue, *E’*) without signs of regurgitation. (*F–G’*) Examples of *alk5a^–/–^* zebrafish exhibiting a detectable aortic flow (*F*) and retrograde blood flow (*F’–G’*). The colour of each dot refers to the same zebrafish across all graphs and images. The dot adjacent to the number symbol (#) identifies the individual zebrafish mentioned in the text. Scale bars: 1 mm (*E–G’*).

Echocardiography, the fastest (approximately 5 minutes per animal) and least invasive imaging technique, records haemodynamic parameters in live animals.^82–85^ We analysed cardiac function from sagittal views of the heart (Supplementary material online, *Figure S2A*), and through Doppler echocardiography were able to assess a range of features including the direction, amount, and velocity of blood flow (*Figure 2A–D* and Supplementary material online, *Figure S2B–K*, *Table 5*). Due to the intrinsic variability between individuals, the heart rate ranged from 58 to 143 beats per minute (bpm), with no statistically significant difference between the two genotypes (*Figure 2A*). Likewise, previous studies have reported heart rate values for WT adult zebrafish ranging from 68 to over 175 bpm.^48,51,53–55^ Regarding haemodynamics, as previously described by our group,^11,86^ we also aimed to assess the presence of blood regurgitation. Therefore, we measured the area of blood inflow and outflow on a two-dimensional (2D) plane and noticed that while most zebrafish exhibited only unidirectional inflow (red; 7/10 in WT and 6/12 in *alk5a*^*–/–*^; *Figure 2C and E*, Supplementary material online, *Video S1*) and outflow (blue; 8/10 in WT and 5/12 in *alk5a*^*–/–*^; *Figure 2D* and *E’*, Supplementary material online, *Video S1*), some *alk5a*^–/–^ zebrafish (7/12) also presented blood flow regurgitation in the atrioventricular canal (AVC; *Figure 2C, F’,G*, Supplementary material online*, Videos S2* and *S^3^*) and/or in the OFT canal (*Figure 2D,G’*, Supplementary material online, *Video S3*). In addition, while most zebrafish exhibited a flow signal exclusively in the heart, a few displayed a signal rostral to the OFT, in the region of the ventral aorta, hereafter referred to as aortic flow (Supplementary material online, *Video S2*). The presence of aortic flow was detected more frequently in *alk5a*^*–/–*^ zebrafish (1/10 in WT and 4/12 in *alk5a*^*–/–*^; *Figure 2B,F*), suggesting an increase in blood flow in their aorta. Nonetheless, the relative inflow and outflow areas in a 2D plane, as well as most haemodynamic parameters including blood flow velocity and ejection time, were highly variable and not significantly different between WT and *alk5a*^–/–^ zebrafish (Supplementary material online, *Figure S2D–K*). In contrast, by determining several parameters in each animal, we identified one *alk5a*^–/–^ zebrafish (indicated by # in all plots) with a large inflow area (Supplementary material online, *Figure S2J*) that also displayed severe blood regurgitation in its AVC and OFT (*Figure 2C,D*), as well as reduced blood flow velocity through its OFT (Supplementary material online, *Figure S2D, E, G*).

**Table 5:** Echocardiography parameters

	Wild type (WT)				*alk5a* ^–/–^				
Parameters	Median	Minimum	Maximum	Inter-quartile range (IQR)	Median	Minimum	Maximum	Inter-quartile range (IQR)	*P*-value
OFT VTI (mm)	12.3	6.9	28.5	11.2–19.4	11.8	1.8	20.3	9.2–16.4	0.2997
OFT mean velocity (mm/s)	77.4	44.7	132.0	64.4–94.9	75.2	17.6	123.5	59.6–95.3	0.5757
OFT mean gradient (mm Hg)	0.024	0.008	0.070	0.017–0.036	0.022	0.001	0.062	0.014–0.037	0.5903
OFT peak velocity (mm/s)	110.4	59.7	186.1	86.6–128.9	98.9	25.6	171.8	82.4–129.4	0.5278
OFT peak gradient (mm Hg)	0.050	0.014	0.139	0.030–0.067	0.040	0.003	0.119	0.027–0.066	0.5229
Ejection time (s)	0.182	0.136	0.246	0.159–0.200	0.157	0.103	0.237	0.137–0.181	0.1947
Heart rate (bpm)	107.8	58.4	131.5	87.4–118.9	98.4	61.0	143.5	84.8–112.3	0.8138
Area aortic flow (×10^3^ mm^2^)	0	0	0.67	0	0	0	3.79	0–0.161	0.1729
Area inflow (×10^4^ AU)	0.81	0.45	1.24	0.67–0.97	0.52	0.04	3.69	0.38–0.82	0.1583
Area outflow (×10^3^ AU)	6.40	3.27	11.75	5.22–6.89	4.12	0.56	10.09	3.47–5.90	0.1447
Regurg. fraction AVC (%)	0	0	11.2	0–1.2	2.6	0	84.0	0–27.0	0.1732
Regurg. fraction OFT (%)	0	0	24.4	0	6.0	0	55.1	0–16.0	0.0847

In summary, despite the great variability obtained with echocardiography, this technique provides important information on cardiac function, particularly regarding haemodynamics in the heart and adjacent vessels.

### 3.3 *In vivo* MRI analyses of beating hearts reveal the expansion of most *alk5a*^–/–^ outflow tracts

To achieve higher resolution of cardiac morphology and performance, we used *in vivo* MRI to study the same 22 zebrafish. MRI is the gold standard technique for clinical analyses of cardiac morphology and function due to its precision and high reproducibility.^87–92^ Although MRI has been increasingly used in small animal models including mice,^93–95^ in zebrafish it has been mostly restricted to *ex vivo* samples or static images of the regenerating heart,^32,33,43,44,96^ without presenting videos of beating hearts. In rodent models, MRI permits the reconstruction of beating hearts,^73,97^ but this type of analysis is challenging in adult zebrafish due to the small size of their heart. Hence, we developed a protocol for adult zebrafish MRI to assess cardiac function in live animals more accurately (see Materials and Methods).

**Figure 3:**
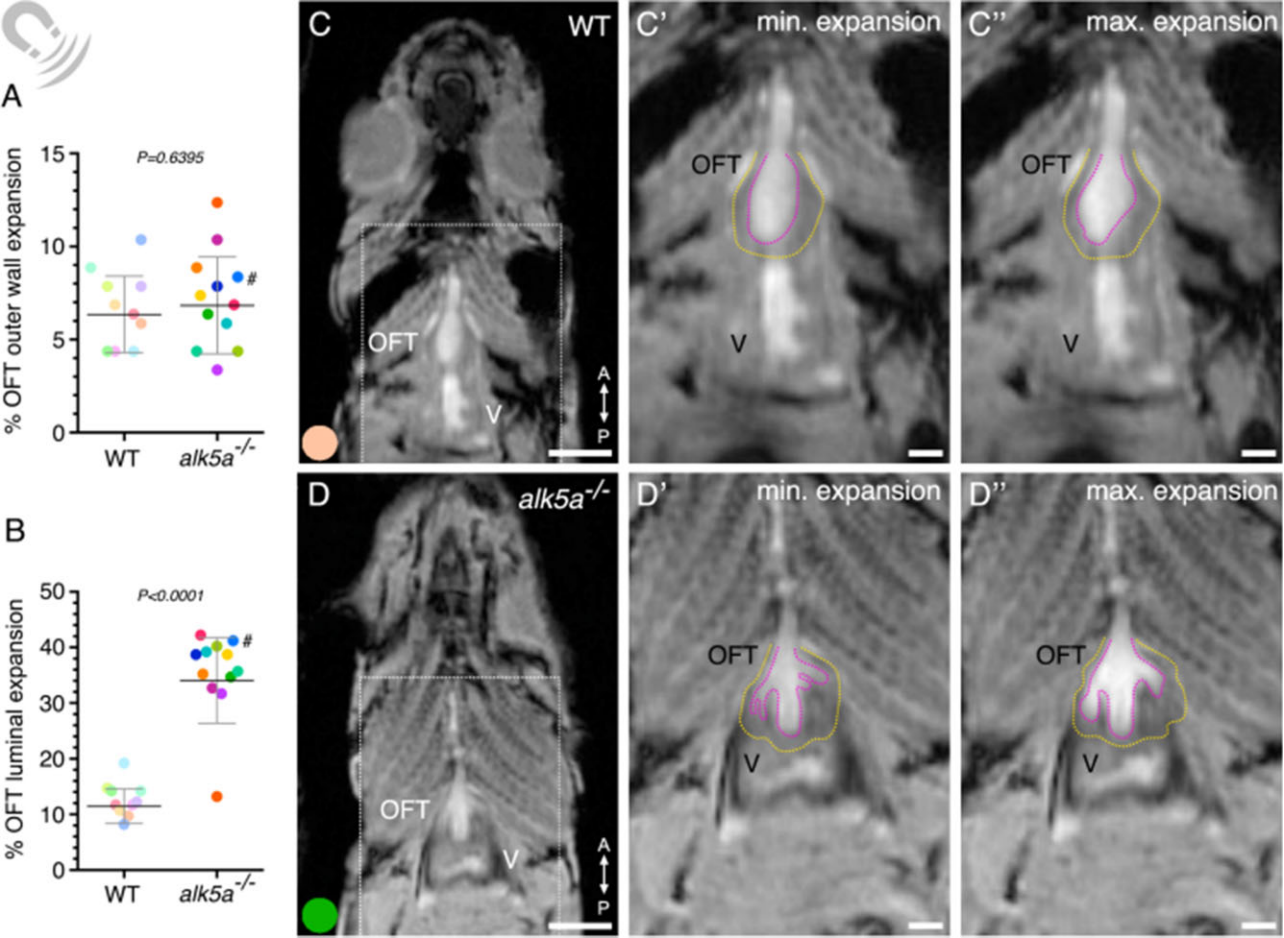
MRI analyses of beating hearts reveal increased OFT luminal dilation in *alk5a^–/–^* adult zebrafish. (*A, B*) Percentage of OFT outer wall (*A*) and luminal (*B*) expansion in WT (*n* = 10) and *alk5a^–/–^* (*n* = 12) adult zebrafish. Plots show the values for each individual and the mean ± SD; *P*-values were determined by unpaired *t*-test (A) or Mann–Whitney test (*B*). (*C–D”*) Single frames of MRI cines of WT (*C–C”*) and *alk5a^–/–^* (*D–D*”) zebrafish in coronal view. Boxed area is shown in *C’, D’* (minimum expansion OFT) and *C”, D”* (maximum expansion OFT). Magenta dashed line, OFT lumen; yellow dashed line, OFT outer wall. The colour of each dot refers to the same zebrafish across all graphs and images. The dot adjacent to the number symbol (#) identifies the individual zebrafish mentioned in the text. OFT, outflow tract; V, ventricle. Scale bars: 1.5 mm (*C, D*), 500 µm (*C’, C”, D’, D”*).

We imaged the heart in two different planes to obtain coronal views of the OFT and ventricle and observe the expansion of these compartments during the cardiac cycle (Supplementary material online, *Figure S3 A–B’*). Additionally, using self-gating signals,^73,97^ we were able to monitor the cardiac rate of the animal (Supplementary material online, *Figure S3 C–C”*) to accurately reconstruct the cardiac cycle. Then, we calculated the percentage of the cardiac chambers’ expansion, measuring both the outer wall and the lumen (*Figure 3A–D”* and Supplementary material online, *Figure S3 D–G*”, *Table 6*). We found that the expansion of the ventricular outer wall and lumen during the cardiac cycle was comparable between WT and *alk5a*^*–/–*^ zebrafish (Supplementary material online, *Figure S3D,E*, Supplementary material online, *Videos S4 and S5*). In contrast, we observed that *alk5a* mutants displayed a severe and consistent increase in OFT luminal dilation (*Figure 3B, C–D”*, Supplementary material online, *Videos S6 and S7*), whereas the OFT outer wall expansion was comparable with that of WT (*Figure 3A,C–D”*). Also, when analysing the results for the *alk5a* mutant with severe haemodynamic defects detected with echocardiography (#), we observed that it had one of the highest OFT luminal expansion levels (*Figure 3B*).

**Table 6:** MRI parameters

	Wild type (WT)				*alk5a* ^–/–^				
Parameters	Median	Minimum	Maximum	Inter-quartile range (IQR)	Median	Minimum	Maximum	Inter-quartile range (IQR)	*P*-value
Vent. lum. exp. (%)	27.3	8.0	59.0	17.6–31.8	28.3	17.5	63.0	20.9–36.4	0.5277
Vent. outer wall exp. (%)	9.0	2.5	22.0	3.1–15.0	6.5	1.0	20.5	4.4–12.6	0.8107
OFT lum. exp. (%)	10.8	7.0	18.0	9.8–13.0	36.0	12.0	41.0	33.0–38.3	<0.0001
OFT outer wall exp. (%)	6.3	4.0	10.0	4.4–7.5	6.8	3.0	12.0	5.1–8.1	0.6395

In summary, by optimizing MRI for beating adult zebrafish hearts, we were able to obtain unique and valuable data on cardiac performance *in vivo*. Moreover, we identified the OFT as the main cardiovascular compartment affected in *alk5a*^*–/–*^ zebrafish.

### 3.4 Micro-CT analyses reveal outflow tract defects in *alk5a*^*–/–*^ adult zebrafish

X-ray-based *ex vivo* CT is considered the most powerful technique to provide high spatial resolution of small structures.^98–101^ Here we use µ**-**CT, which is capable of volumetric analysis with a voxel size <20 µm,^102–104^ for a novel characterization of the 3D morphology of the adult zebrafish cardiac compartments and adjacent vessels. Due to the exposure to radiation, the requirement for a contrast agent, and for long imaging periods to acquire high-resolution images, we performed this imaging on fixed samples. Individual X-ray projections were used to reconstruct cross-section images, providing views of the tissues in multiple planes (Supplementary material online, *Figure S4A–A”*).

**Figure 4:**
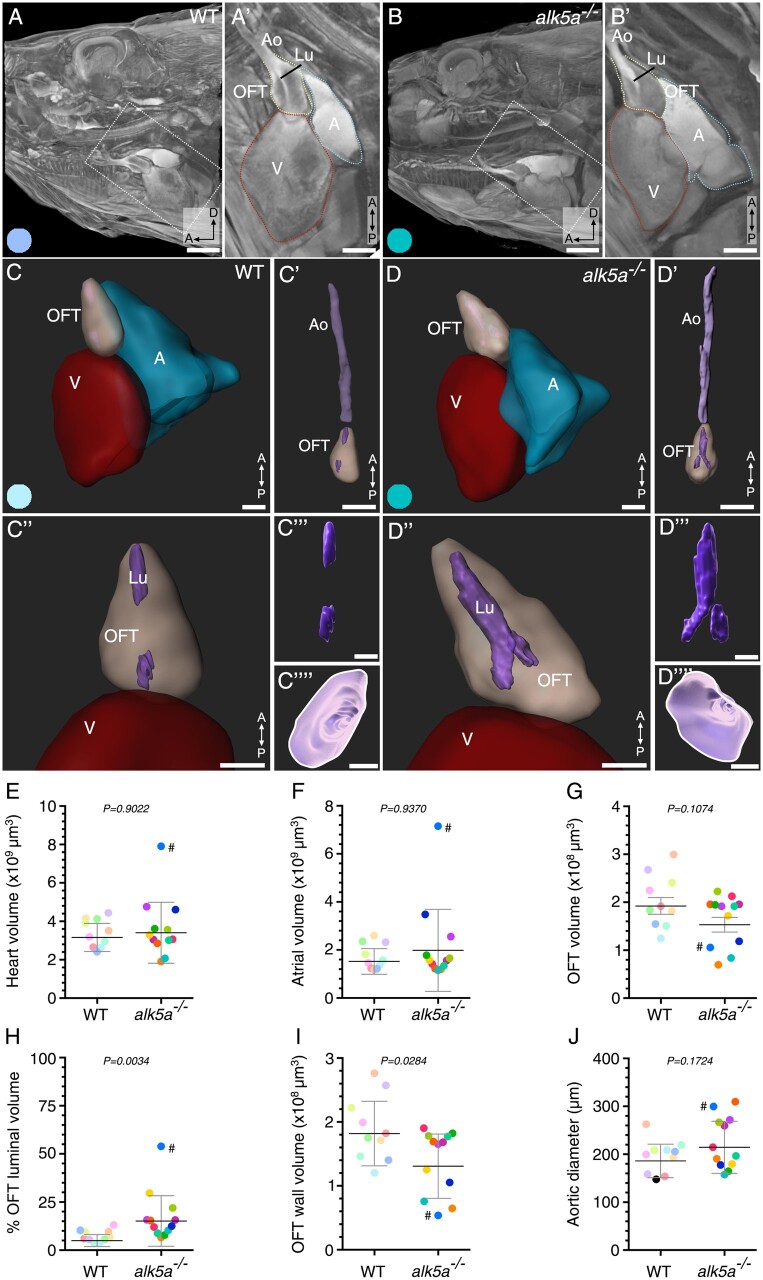
µ-CT analyses of cardiovascular morphology in *alk5a^–/–^* adult zebrafish reveal OFT defects. (*A, B*) µ-CT scans showing a sagittal plane of the anterior region of WT and *alk5a^–/–^* adult zebrafish. Boxed area is shown in A’ and B’. Dashed lines outline cardiac compartments. (*C–D””*) 3D reconstructions of the cardiac compartments in WT (*C–C””*) and *alk5a^–/–^* (*D–D””*) zebrafish, including the aorta (C’, D’) and OFT lumen (Lu; *C’”, D’”*). (*C””, D””*) 3D reconstructions of the aortic opening (white line) as seen from the OFT. (*E–J*) Quantification of morphological parameters for each cardiac compartment in WT (*n* = 10) and *alk5a^–/–^* (*n* = 12) zebrafish. Plots show the values for each individual and the mean ± SD; *P*-values were determined by unpaired *t-*test (*G–J*) or Mann–Whitney test (*E, F*). The colour of each dot refers to the same zebrafish across all graphs and images. The dot adjacent to the number symbol (#) identifies the individual zebrafish mentioned in the text. A, atrium; Ao, aorta; OFT, outflow tract; V, ventricle. Scale bars: 1 mm (*A, B*), 500 µm (*A’, B’*), 300 µm (*C, D’*), 400 µm (*C”–D”*”).

After manual segmentation and volumetric surface rendering (*Figure 4A–D””*, Supplementary material online, *Videos S8*–*S11*), we could determine the volume of each cardiac compartment, as well as the diameter and volume of the aorta (*Figure 4E–J* and Supplementary material online, *Figure S4 B–G*, *Table 7*, Supplementary material online, *Videos S12 and S13*). Consistent with the measurements obtained with the other techniques, we did not observe any statistically significant differences between WT and *alk5a*^*–/–*^ zebrafish regarding the volume of the entire heart (*Figure 4E*), atrium (*Figure 4F*, Supplementary material online, *Figure S4D*), or ventricle (Supplementary material online, *Figure S4B* and *C*). Moreover, while the total volume of the OFT appeared to be lower in *alk5a*^*–/–*^ zebrafish, this difference was not statistically significant (*Figure 4G* and Supplementary material online, *Figure S4E*). However, *alk5a*^*–/–*^ zebrafish displayed a statistically significant increase in OFT luminal volume (relative to OFT volume; *Figure 4H*), and a decrease in OFT wall volume (*Figure 4I*). Despite the observation of aortic flow in *alk5a*^–/–^ zebrafish suggesting higher aortic volumes, *alk5a*^*–/–*^ zebrafish exhibited only a slight increase in aortic diameter compared with WT (*Figure 4C””*,*D””*,*J*, Supplementary material online, *Videos S14 and S15*), while displaying an unaltered volume (Supplementary material online, *Figure S4G*).

**Table 7:** Micro-CT parameters

	Wild type (WT)				*alk5a* ^–/–^				
Parameters	Median	Minimum	Maximum	Inter-quartile range (IQR)	Median	Minimum	Maximum	Inter-quartile range (IQR)	*P*-value
Heart vol. (×10^9^ µm^3^)	3.11	2.18	4.20	2.49–3.84	2.95	1.67	7.67	2.74–3.62	0.9022
Vent. vol. (×10^8^ µm^3^)	1.40	1.00	2.00	1.19–1.75	1.41	6.07	1.99	9.28–10.53	0.3142
Vent. vol. (%)	46.00	34.20	61.00	43.3–49.5	46.00	8.10	53.50	41.7–49.6	0.9094
Atrial vol. (×10^9^ µm^3^)	1.33	9.91	2.41	1.08–2.01	1.38	9.68	6.96	1.12–1.78	0.9370
Atrial vol. (%)	48.30	30.20	61.40	43.4–51.0	48.00	39.40	90.70	43.8–54.4	0.7354
OFT vol. (×10^8^ µm^3^)	1.78	1.15	2.90	1.52–2.27	1.82	6.02	2.13	1.06–1.86	0.1074
OFT vol. (%)	6.20	4.40	8.80	4.9–7.1	5.40	1.30	7.20	3.9–6.6	0.1328
OFT lum. vol. (×10^7^ µm^3^)	0.78	0.16	2.30	0.59–1.50	2.19	0.25	4.95	1.08–2.90	0.1328
OFT lum. vol. (%)	4.00	1.10	10.70	0.3–0.7	11.60	4.20	51.50	7.5–14.9	**0.0034**
OFT wall vol. (×10^8^ µm^3^)	1.72	1.13	2.69	1.46–2.09	1.59	0.47	1.83	0.91–1.71	**0.0284**
Aortic diam. (µm)	193.00	138.00	253.00	157.8–199.0	196.00	148.00	300.00	169.5–258.3	0.1724
Aortic vol. (×10^7^ µm^3^)	4.06	2.17	7.73	3.31–6.46	4.09	1.55	9.64	3.18–6.63	0.8638

**Table 8:** Regurgitation fraction values obtained in the sequential echocardiography measurements (related to Figure 7)

		WT (%)						*alk5a* ^–/–^ (%)					
		Control			+ISO			Control			+ISO		
	Zebrafish	t1	t2	t3	t1	t2	t3	t1	t2	t3	t1	t2	t3
Regurgitation fraction OFT	1	0.0	0.0	0.0	0.0	0.0	0.0	0.0	0.0	0.0	0.0	1.9	11.6
	2	0.0	0.0	0.0	0.0	0.0	0.0	0.0	0.0	7.9	51.9	39.4	83.1
	3	0.0	0.0	0.0	0.0	0.0	0.0	0.7	0.0	0.0	0.0	0.0	0.0
	4	0.0	0.0	0.0	0.0	0.0	0.0	90.7	100.0	100.0	0.0	0.0	0.9
	5	0.0	0.0	0.0	0.0	0.0	0.0	0.0	0.0	0.0	23.9	44.9	28.3
	6	0.0	0.0	0.0	0.0	0.0	0.0	0.0	0.0	0.0	0.0	0.0	0.0
	7	0.0	0.0	0.0	0.0	0.0	0.0				0.0	0.0	2.2
Regurgitation fraction AVC	1	1.2	0.0	0.0	0.0	0.0	0.0	3.3	0.0	0.0	0.0	0.1	21.4
	2	1.4	0.7	0.0	0.0	0.0	0.0	2.0	0.8	21.5	0.0	0.0	1.4
	3	0.0	0.0	0.0	0.0	0.0	0.0	0.0	0.0	0.0	56.7	39.2	68.7
	4	15.3	0.0	0.0	12.4	0.0	12.5	100.0	100.0	100.0	0.0	39.4	40.7
	5	0.0	0.0	0.0	45.2	7.2	1.5	0.0	0.0	0.0	0.0	0.0	0.0
	6	0.0	0.0	0.0	0.0	0.0	2.1	30.7	4.1	55.4	0.0	0.0	76.3
	7	0.0	0.0	0.0	0.0	0.0	6.9				0.0	3.0	0.5

It is important to note that the µ-CT analyses provided sufficient resolution to visualize smaller structures such as the ventricular trabeculae (*Figure 4A–B’* and Supplementary material online, *Figure S4A”*) and the atrioventricular valve (*Figure 4B’*). Moreover, by tracking individual zebrafish with the different techniques, we observed that the zebrafish displaying the most severe functional phenotype as assessed by echocardiography and MRI (#) also presented severe morphological defects when analysed with µ**-**CT. In particular, this individual exhibited the largest OFT luminal volume (*Figure 4H*) and one of the largest aortic diameters (*Figure 4J*), but the smallest OFT wall volume (*Figure 4I*). This zebrafish also displayed an abnormally large atrial volume (*Figure 4F*), in line with the high regurgitation fraction in the AVC identified by echocardiography.

Thus, with the help of µ**-**CT imaging, we were able to obtain precise structural information about all the cardiac compartments and further resolve the OFT defects identified using the other platforms.

### 3.5 Correlation analysis between all parameters reveals previously undetected phenotypes in *alk5a*^*–/–*^ adult zebrafish

**Figure 5:**
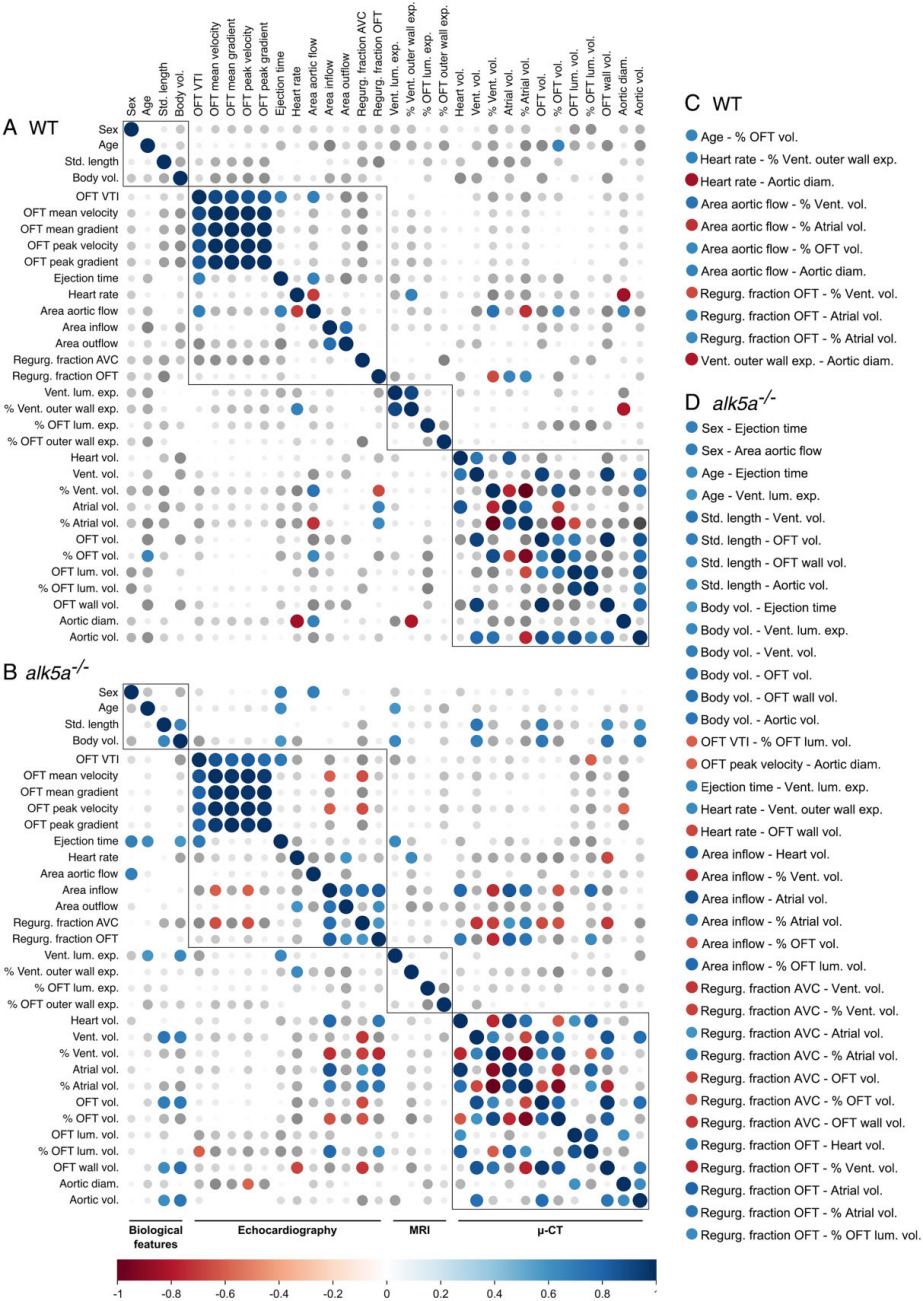
Correlation analysis of all the measured parameters reveals previously undetected cardiovascular phenotypes. (*A, B*) Correlograms of all the parameters analysed including biological features, as well as echocardiography, MRI and μ-CT analyses across all the WT (*A*; *n* = 10) and *alk5a^–/–^* (*B*; *n* = 12) zebrafish. Positive and negative correlations are shown in blue and red, respectively; dot size represents Pearson’s correlation coefficient. (*C, D*) Significant correlations (*P* < 0.05) in WT (*C*) and *alk5a^–/–^* (*D*) zebrafish.

From the 32 parameters measured with the different imaging platforms, only three showed a *P* < 0.05 between WT and *alk5a*^–/–^ samples after bulk analysis. The lack of statistical significance in group comparisons is due to the intrinsic variability between individuals of the same genotype, a factor which needs to be considered when analysing heterogeneous samples. However, when one follows a single individual (e.g. #), it becomes clear that a higher severity in functional phenotypes is accompanied by stronger morphological alterations. Therefore, we analysed how variables were linked by determining the pairwise Pearson’s correlation coefficients across all the WT zebrafish and across all the *alk5a*^–/–^ zebrafish, herein represented as correlograms (WT, *Figure 5A*, and *alk5a*^*–/–*^*, Figure 5B*).

As expected, we observed strong correlations between measurements obtained within the same platform, but also several interesting inter-platform correlations. As seen in humans where cardiovascular parameters strongly correlate with gender, age and ethnicity,^38,92,105–107^ we noticed within the WT group a positive correlation between age and the volume of the OFT relative to the entire heart (% OFT volume; *Figure 5C*). Furthermore, retrograde OFT flow, which was present in two WT zebrafish, correlated with atrial enlargement.

In addition to the 11 correlations observed in the WT zebrafish, we identified 37 new correlations in the *alk5a* mutants (*Figure 5B and D*), suggesting a disruption of the morphology and/or function of the mutant heart. For example, we were able to address whether defects in OFT morphology affect cardiac performance and haemodynamics and vice versa. Indeed, we identified a negative correlation between OFT wall volume and heart rate, as well as between OFT luminal volume relative to the OFT (% OFT luminal volume) and OFT velocity time integral (VTI), suggesting that OFT morphology is linked with cardiac performance and blood velocity. We also observed a negative correlation between OFT volume and OFT wall volume with the regurgitation fraction in the AVC, suggesting that a more pronounced AVC retrograde flow is linked with defective (smaller and thinner) OFTs. Furthermore, there was a positive correlation between % OFT luminal volume and regurgitation fraction in the OFT, suggesting that larger OFT lumens are associated with higher retrograde flow in the OFT. When analysing whether these morphological OFT defects were influenced by age or body size, we found that OFT total and wall volumes were negatively correlated with standard length and body volume, suggesting an aggravation of these phenotypes in older and bigger zebrafish.

Regarding other cardiac compartments, the extent of retrograde flow across the AVC was associated with a smaller ventricle and bigger atrium, consistent with the consequences of valve regurgitation which leads to a higher amount of blood stalling in the atrium.^108^ Interestingly, *alk5a*^*–/–*^ zebrafish—but not WT—with a higher regurgitation fraction in the OFT also displayed higher regurgitation fraction in the AVC, suggesting that these defects are closely associated, at least in the *alk5a* mutants.

These correlation analyses helped us to identify links between morphological and functional phenotypes, even when statistical tests comparing WT and mutant groups failed to provide significance due to the high variability of the defects.

Additionally, in order to better understand the cardiovascular phenotypes, we tested whether *alk5a*^*–/–*^ adult zebrafish displayed electrophysiological defects using electrocardiography (ECG) analysis (Supplementary material online, *Figure S5*). Because we lacked the required expertise in house, these studies were performed in a different site and with a different set of zebrafish. We were able to identify arrhythmia in four of the six *alk5a*^*–/–*^ zebrafish analysed while no *alk5a^+/+^* siblings exhibited any obvious defects (Supplementary material online, *Figure S5A–B’*). There were no statistically significant differences in P-wave duration, PR interval (representing atrial depolarization), or QRS interval (representing ventricular depolarization and contraction) between *alk5a^+/+^* and *alk5a*^*–/–*^ zebrafish (Supplementary material online, *Figure S5A”–E*). However, *alk5a* mutants exhibited higher variability in P-wave duration and PR interval in the same individual (Supplementary material online, *Figure S5A”,B”*) and between individuals (Supplementary material online, *Figure S5C,D*), suggesting alterations of the conduction system and setting the ground for future studies.

### 3.6 Combined imaging analyses facilitate the selection of specific functional phenotypes for downstream morphological characterization

**Figure 6:**
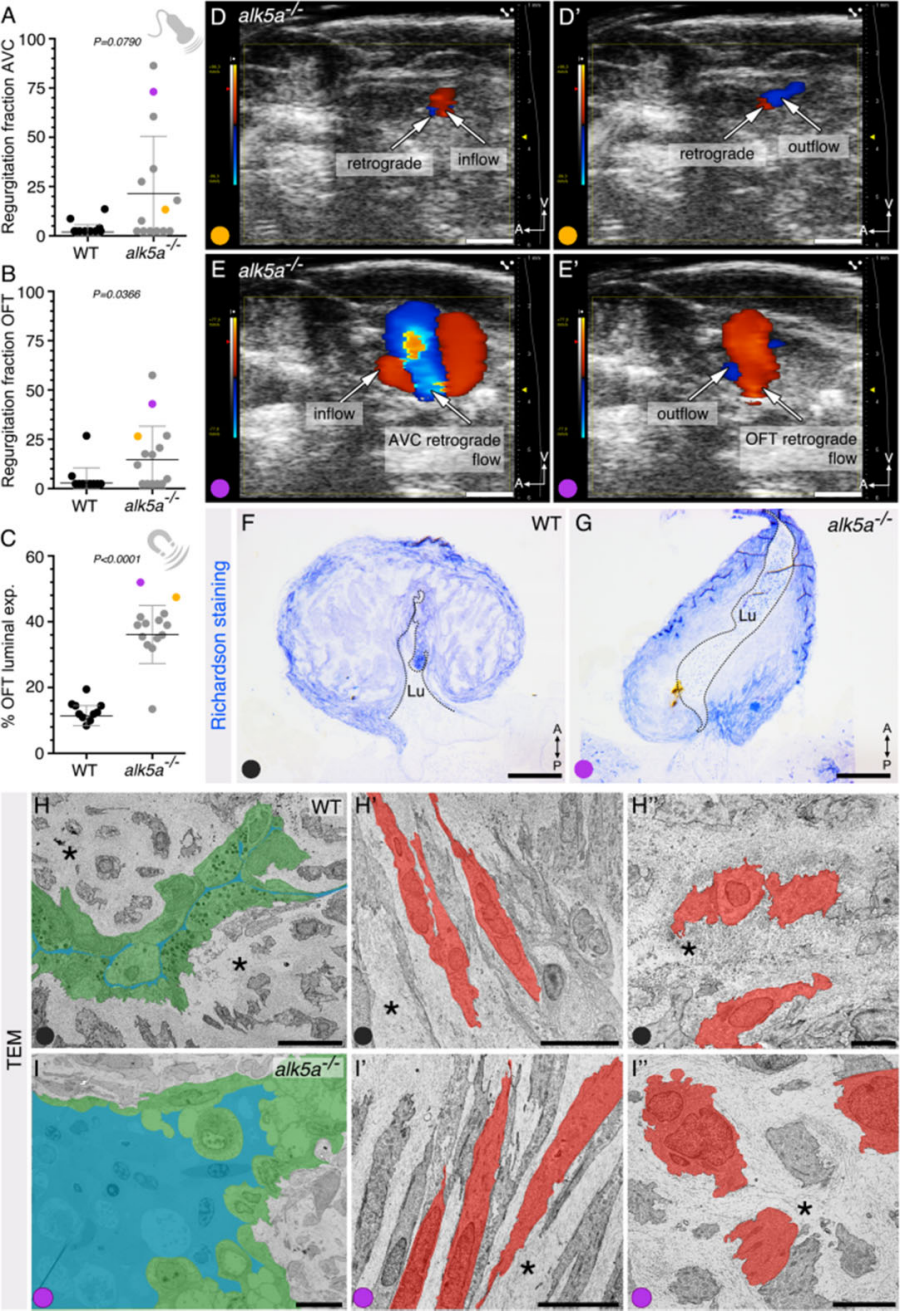
Combined imaging analyses facilitate the selection of specific phenotypes for high resolution morphological characterization. (*A–C*) Regurgitation fraction as obtained by echocardiography (*A, B*) and OFT luminal expansion as obtained by MRI (*C*) for two zebrafish (orange and purple, *n* = 2) plotted against the previously measured zebrafish (WT, black, *n* = 10; *alk5a^–/–^* grey, *n* = 12). Plots show the values for each individual and the mean ± SD; *P*-values were determined by Mann–Whitney test. Purple dot identifies the zebrafish selected for subsequent analysis with transmission electron microscopy (TEM). (*D–E’*) *alk5a^–/–^* zebrafish showing mild (orange, *D, D’*) and severe (purple, *E, E’*) regurgitation fraction in the AVC and OFT regions. (*F, G*) Semi-thin sections of WT and *alk5a^–/–^* OFTs stained with Richardson staining solution. Dashed line outlines the OFT lumen (Lu). (*H–I”*) TEM images of WT and *alk5a^–/–^* OFTs, showing the OFT lumen in blue, lined by ECs in green (H, I) and the OFT wall (*H’, H”, I’, I”*), including SMCs (red) and ECM (asterisk). Scale bars: 1 mm (*D–E’*), 200 µm (*F, G*), 5 µm (*H*), 10 µm (*H’–I”*).

Having observed that *alk5a*^*–/–*^ zebrafish frequently display retrograde blood flow and increased expansion of their OFT lumen, we analysed additional animals using echocardiography and MRI to perform further analyses (*Figure 6A–E’*). We chose an *alk5a*^*–/–*^ zebrafish displaying severe retrograde blood flow through its AV canal (*Figure 6A,D,E*), OFT blood regurgitation (*Figure 6B,D’,E’*), as well as an unusually large expansion of its OFT lumen (*Figure 6C*). To analyse the morphological and ultrastructural defects in this animal, we performed light microscopy (*Figure 6F,G*) and transmission electron microscopy (TEM; *Figure 6H–I”*) on tissue sections. Semi-thin sections for light microscopy showed a dilated lumen and thinner wall in the OFT (*Figure 6F,G*). TEM analysis revealed that the lumen of the WT OFT was barely detectable and lined with flattened or cuboidal endothelial cells (ECs; *Figure 6H*). We also observed that the WT OFT wall was composed of both spindle-shaped and cuboidal smooth muscle cells (SMCs) surrounded by a dense ECM (*Figure 6H’,H”*). In contrast, the mutant OFT displayed an enlarged lumen lined by rounded ECs (*Figure 6I*). Notably, many cells lining the lumen in *alk5a*^*–/–*^ OFT appeared empty or detached, suggesting cell death. Despite a thinner wall and a sparser and less dense ECM in the *alk5a*^*–/–*^ OFT, there were no obvious alterations in the SMCs (*Figure 6I’,I”*).

Overall, these data indicate that non-invasive imaging techniques such as echocardiography and *in vivo* MRI represent useful platforms to identify individuals displaying functional phenotypes (*Table 9*) for further downstream high-resolution analyses.

**Table 9:** Summary of the cardiovascular defects identifiable with each imaging technique

	Extracted data	Strengths	Weaknesses	Phenotypes
Histological sections	Tissue morphology and cellular organization	High resolution Identification of different cell types Marker analyses (e.g. immunostaining) Identification of tissue alterations	Static images 2D assessment Need to sacrifice the animal Loss of anatomical information due to tissue extraction	Alterations at the cellular level (e.g. cell specification, cell death, cell proliferation) Alterations at the tissue level (e.g. tissue morphology, inter-tissue organization)

Echocardiography	Haemodynamic parameters (blood velocity and direction) 2D assessment of ventricular size Heart rate	*In vivo* data recording Only technique to assess haemodynamic parameters Monitoring the progression of phenotypes Fast imaging	Low tissue resolution 2D assessment	Altered blood flow due to impaired cardiac pumping capacity Cardiac valve malfunction leading to blood regurgitation Abnormal blood flow in the great vessels Severe alterations of the ventricular size—assessment in 2D Severe arrhythmia

*In vivo* MRI	Contraction/expansion of cardiac compartments 2D assessment of ventricular size Heart rate	*In vivo* data recording Sufficient resolution to assess cardiac chambers expansion Monitoring the progression of phenotypes	Low tissue resolution 2D assessment Heart rate based on indirect measurement Slow imaging acquisition Complex and expensive set up	Altered contraction/expansion of cardiac compartments Gross morphological alterations of the cardiac compartments—assessment in 2D Severe arrhythmia

*Ex vivo* µ-CT	Volume of the cardiac compartments Spatial organization of the cardiac Gross morphological features of the heart, connecting vessels and cardiac valves	Preserves heart integrity– no need for organ extraction High resolution at the tissue level Provides spatial information of the heart and surrounding tissues (3D) Data on morphology of connecting vessels	Static images *Ex vivo* analyses—need for animal sacrifice Time-consuming analysis Expensive	Altered volumes of cardiac compartments (static) – assessment in 2D and/or 3D Morphological alterations of the great vessels Gross morphological alterations of the myocardium and cardiac valves Abnormal spatial position/organization of the cardiac compartments and accessory vessels

ECG	Electrophysiology of the heart Heart rate Heart rhythm	Monitoring the progression of phenotypes Fast imaging	No tissue observation	Altered cardiac conduction Arrhythmia

### 3.7 Characterizing phenotypic progression using sequential *in vivo* measurements

**Figure 7:**
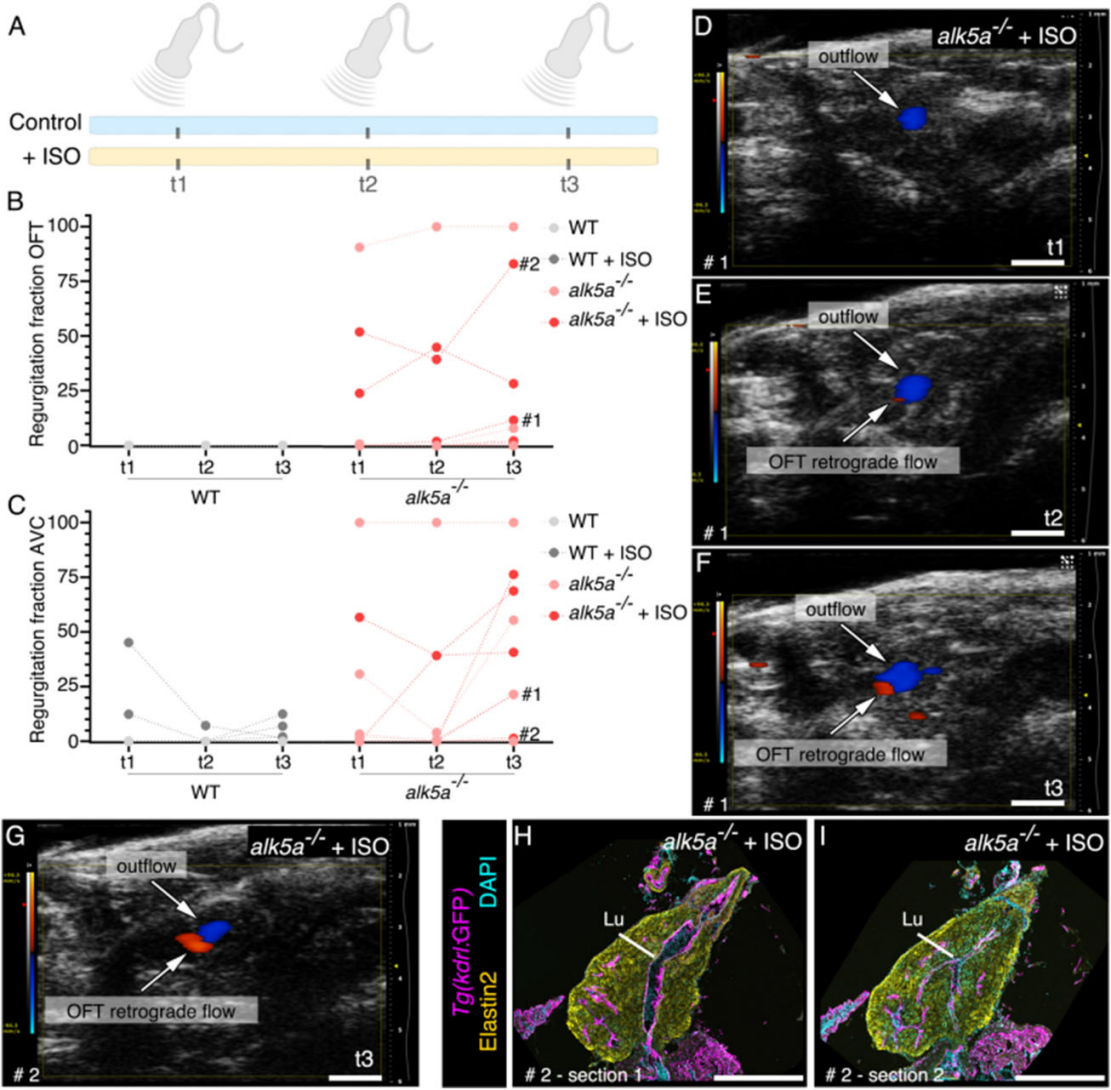
Sequential *in vivo* measurements allow the observation of phenotype progression. (*A*) Experimental setup for the sequential monthly measurements (t1, t2, t3) performed with echocardiography in WT and *alk5a^–/–^* in control conditions (*n* = 7 WT, *n* = 6 *alk5a^–/–^*) and upon treatment with isoprenaline (+ISO; *n* = 7 WT, *n* = 7 *alk5a^–/–^*). (*B–F*) Graphs showing changes in OFT (*B*) and AVC (*C*) regurgitation in WT and *alk5a^–/–^* adult zebrafish untreated or treated with isoprenaline. (*G–I*) Zebrafish # 2, which displays the most pronounced increase in the regurgitation fraction in the OFT from t1 to t3 (*G*), presented an enlarged OFT lumen in some histological sections (*H*) but not in others (*I*). Scale bars: 1 mm (*D–G*), 400 µm (*H, I*).

In order to determine whether we could analyse the progression of the *alk5a*^*–/–*^ phenotype in the same zebrafish, we decided to use echocardiography, as the least invasive technique, to follow the same zebrafish in three measurements at 0 (t1), 3 (t2), and 8 (t3) weeks (*Figure 7*; *Table 8*). Interestingly, we observed that some zebrafish with OFT retrograde flow consistently presented this defect at all three time-points, while others never did, further illustrating the robustness of the method.

Additionally, we treated both WT and *alk5a*^*–/–*^ zebrafish with isoprenaline to increase the heart rate,^69–72^ in order to determine whether we could aggravate the phenotype, or accelerate its progression in *alk5a*^–/–^ zebrafish (*Figure 7A*). We observed that in WT zebrafish the regurgitation fraction in the OFT and AVC was very low, or none, at all time-points, and even after isoprenaline treatment; however, in some *alk5a*^*–/–*^ zebrafish, we could observe an increase in the regurgitation fraction from t1 to t3 (*Figure 7B–F*; *Table 8*). Most of the *alk5a*^*–/–*^ zebrafish treated with isoprenaline (5/7) exhibited no detectable functional phenotype at the beginning of the experiment (t1, *Figure 7B,D–F*), but several of them (3/5) progressively started to display blood regurgitation in the OFT at t2 and t3 (*Figure 7E,F*). Notably, at t3, only 2/6 of the untreated *alk5a*^*–/–*^ zebrafish exhibited OFT regurgitation, while 5/7 of the isoprenaline*-*treated *alk5a* mutants did (*Table 8*).

Interestingly, when analysing histological sections of the *alk5a*^*–/–*^ zebrafish with the most severe increase in OFT blood regurgitation during the isoprenaline experiment (zebrafish 2, *Figure 7G–I*), we noticed that only some of the sections showed a dilated OFT lumen, highlighting the importance of a comprehensive analysis.

## 4. Discussion

In this study, we developed new protocols and combined multiple cardiovascular imaging modalities including *in vivo* echocardiography and MRI along with *ex vivo* µ-CT to analyse the adult zebrafish heart. For each animal, we measured 32 different cardiovascular parameters in WT and *alk5a*^–/–^ samples. Bulk analyses of *alk5a*^*–/–*^ vs WT, as well as intra-individual correlations between these parameters led to the identification of a primary OFT phenotype.

### 4.1 Optimization of preclinical cardiovascular imaging analysis in zebrafish

While during development it is possible to use microscopy for single-cell resolution imaging of the zebrafish internal organs, as the animals grow, the tissues become opaque forcing researchers to rely on tissue sections. Previous publications have reported the optimization of preclinical imaging techniques such as echocardiography, MRI, and µ-CT for adult zebrafish. However, these studies have not (i) characterized haemodynamics such as blood regurgitation using echocardiography; (ii) shown functional MR images of reconstructed beating hearts in living animals; (iii) quantified the volumes of the cardiac compartments using µ-CT; and (iv) analysed the integrated data obtained from different techniques.

Here, taking advantage of the echocardiography Colour Doppler Mode, we were able to gather data on the haemodynamics within the heart and aorta of adult zebrafish. We observed blood flow perturbation in some *alk5a*^–/–^ zebrafish including blood regurgitation in the AV and OFT canals, and increased aortic flow.

With our new *in vivo* MRI protocol, we were able to observe the contracting heart with considerably improved image contrast and resolution, compared with echocardiography. This technique allowed us to assess cardiac performance in adult zebrafish, and we observed that 11/12 *alk5a*^*–/–*^ zebrafish exhibited a severely expanded OFT.

We complemented this functional analysis with *ex vivo* µ-CT imaging to examine the morphology of the adult zebrafish heart at high resolution. With a few exceptions,^30,45,46,109,110^ this technique has been mainly used for *ex vivo* samples, and focusing on the skeleton.^34,111–113^ Three dimensional (3D) rendering of the cardiac compartments and aorta allowed the identification of alterations specifically in the OFT, atrial volume, and diameter of the aorta in some *alk5a*^–/–^ zebrafish. Since this imaging modality does not require tissue dissection, the preservation of tissue integrity and organ positioning brings additional information, allowing the investigation of phenotypes affecting the great vessels and other connecting structures. Developing the use of µ-CT for *in vivo* imaging will have to take into consideration the exposure to radiation and the need to immobilize the sample during data acquisition. This approach, also combined with an intra-venous contrast agent, would be especially powerful to study aneurysm development and the progression of conditions affecting cardiac performance, including during cardiac regeneration.

Moreover, much like clinicians strongly rely on reference values to define a healthy heart considering the gender, age and ethnicity of the patients,^38,92,105–107^ we define some reference values for each technique (*Tables* 5–7). We also summarized the information that can be extracted, and the phenotypes that can be analysed, with each technique, highlighting their strengths and weaknesses, in order to help select the optimal approach(es) (*Table 9*).

### 4.2 Multimodal approach to identify cardiovascular phenotypes

When designing studies with adult specimens, the reduced sample size and the high individual variability need to be considered. Moreover, the same disease-causing mutation can lead to a phenotype in only some of the mutant individuals (incomplete penetrance), or lead to different phenotypic severity (variable expressivity).^37^ In our analyses, we observed high phenotypic variability between individuals, particularly related to haemodynamic measurements. Overall, we observed that few of the parameters measured with each technique were significantly different (defined as a *P*-value <0.05) between WT and *alk5a*^–/–^. Nevertheless, it was evident that some *alk5a*^–/–^ zebrafish presented cardiovascular defects. Therefore, in order to investigate this variability between individuals, we subjected the same zebrafish to multiple imaging techniques to increase the data collected per individual and decrease the number of specimens required. We used these techniques to select specimens with specific features for high resolution approaches including TEM. This approach is similar to that proposed by clinicians who have advocated the use of multimodal imaging tools for a more accurate diagnosis of complex and variable cardiovascular diseases.^39,114–116^

In zebrafish thus far, only a few studies have integrated different modalities for phenotypic characterization, for example combining µ-CT and electron microscopy.^112,117^ Here, we analysed parameters that encompass haemodynamics, cardiac performance, and morphology in the same zebrafish. To highlight the power of a sensitive and comprehensive imaging platform, we selected to study a model with a relatively mild cardiovascular phenotype. In fact, only 50% of *alk5a*^–/–^ zebrafish exhibit defects as observed on histological sections, making the study of the phenotype with solely morphological techniques inconclusive. Accordingly, with our imaging platform, we identified some zebrafish exhibiting clear functional defects (as assessed by echocardiography and/or MRI), but lacking obvious morphological defects (as assessed by µ-CT). ECG analysis suggests that *alk5a*^*–/–*^ adult zebrafish also display electrophysiological defects, including cardiac arrhythmia and high variability in atrial depolarization; and, it will be interesting to integrate the ECG into our multimodal platform to complement the image analysis with electrophysiology. Moreover, by correlation analysis of each parameter, in addition to bulk analysis of WT vs mutant samples, and by selecting individuals with specific functional phenotypes, we achieved a better understanding of the cardiovascular phenotypes in *alk5a*^–/–^ zebrafish.

### 4.3 Clinical perspective of the *alk5a* mutant cardiovascular phenotype

Overall, our data indicate that while the loss of *alk5a* alone does not cause embryonic defects,^64^ it can cause cardiovascular defects which often remain asymptomatic even at adult stages. Our data also identify the OFT (orthologous to the mammalian adult aortic root) as the main compartment affected in *alk5a*^–/–^ zebrafish. Many of the phenotypes we observed are similar to those associated with aortopathies in clinical settings, pathologies often derived from mutations in TGF-β pathway genes.^59–61^ At the morphological level, we observed that *alk5a*^–/–^ zebrafish present a thinning of the OFT outer wall, similar to patients affected by aortic aneurysms prone to dissection.^118,119^ It is also interesting to note that the volume of the OFT wall decreased according to the age and size of the zebrafish, similar to the decrease in aortic wall thickness in older patients.^120,121^ At the functional level, we observed that increased OFT dilation was linked with retrograde blood flow, as often reported in patients presenting aortic dilation and regurgitation.^122–125^ In addition, the increased aortic flow phenotype in *alk5a*^–/–^ zebrafish resembles what has been reported in human patients in whom aortic dilation can cause disturbed and tortuous flow not only in the aneurysm site but also in apparently unaffected aortic portions.^126^ Unfortunately, detecting aneurysms in adult zebrafish is challenging due to the limited resolution of the current techniques. However, future implementation of *in vivo* µ-CT with intra-venous contrast agents, and/or the integration of flow measurements with MRI in higher resolution, might help with the detection of such phenotypes.

In summary, we propose a combinatorial imaging and data analysis platform to detect phenotypes that are too variable or subtle to identify with conventional histological analyses. This study further illustrates the importance of the zebrafish model to help clarify adult phenotypes potentially translatable to the clinic.

## Supplementary material

Supplementary material is available at *Cardiovascular Research* online.

## Supplementary Material

cvab310_Supplementary_Data

## Data Availability

The data underlying this article will be shared on reasonable request to the corresponding authors.
