# Supplementary material for: Integration of multiple imaging platforms to uncover cardiovascular defects in adult zebrafish
Source: Cardiovasc Res. 2021 Oct 5;118(12):2665–87. doi: 10.1093/cvr/cvab310 (PMC9491864; doi:10.1093/cvr/cvab310)
Supplement: cvab310_Supplementary_Data [file cvab310_supplementary_data.zip › Supplementary material_final CVR-2020-1278R2.docx]

**Supplementary material**

**Integration of multiple imaging platforms to uncover cardiovascular defects in adult zebrafish**

Anabela Bensimon-Brito^1,2,3^*^#^, Giulia L. M. Boezio^1,2,4^*, João Cardeira-da-Silva^1,2^, Astrid Wietelmann^5^, Srinath Ramkumar^1,2^, Pia R. Lundegaard^6, 7^, Christian S. M. Helker^1,8^, Radhan Ramadass^1^, Janett Piesker^9^, Arno Nauerth^10^, Clemens Mueller^11^, Didier Y. R. Stainier^1,2#^

**
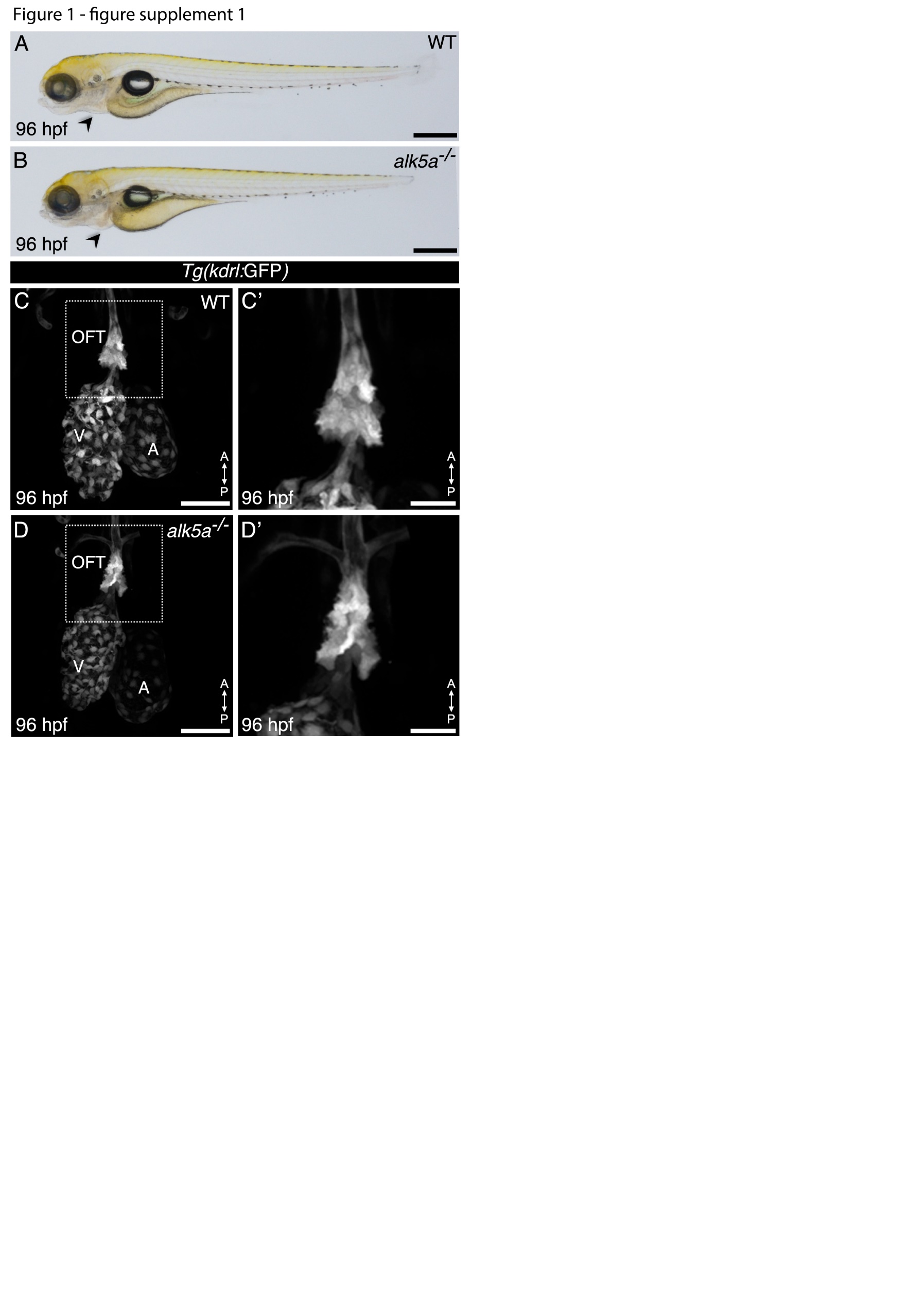
**

**Figure S1 – *alk5a* mutant larvae exhibit no obvious morphological defects.**

(A, B) Brightfield images of 96 hpf WT (A) and *alk5a^-/-^* (B) larvae show no obvious morphological differences. (C-D’) Confocal images of 96 hpf *Tg(kdrl:*GFP*)* WT (C) and *alk5a^-/-^* (D) hearts. Boxed area is shown in C’ and D’. A- atrium, V- ventricle, OFT- outflow tract. Scale bars: 400 μm (A, B), 50 μm (C, D), 20 μm (C’, D’).

**
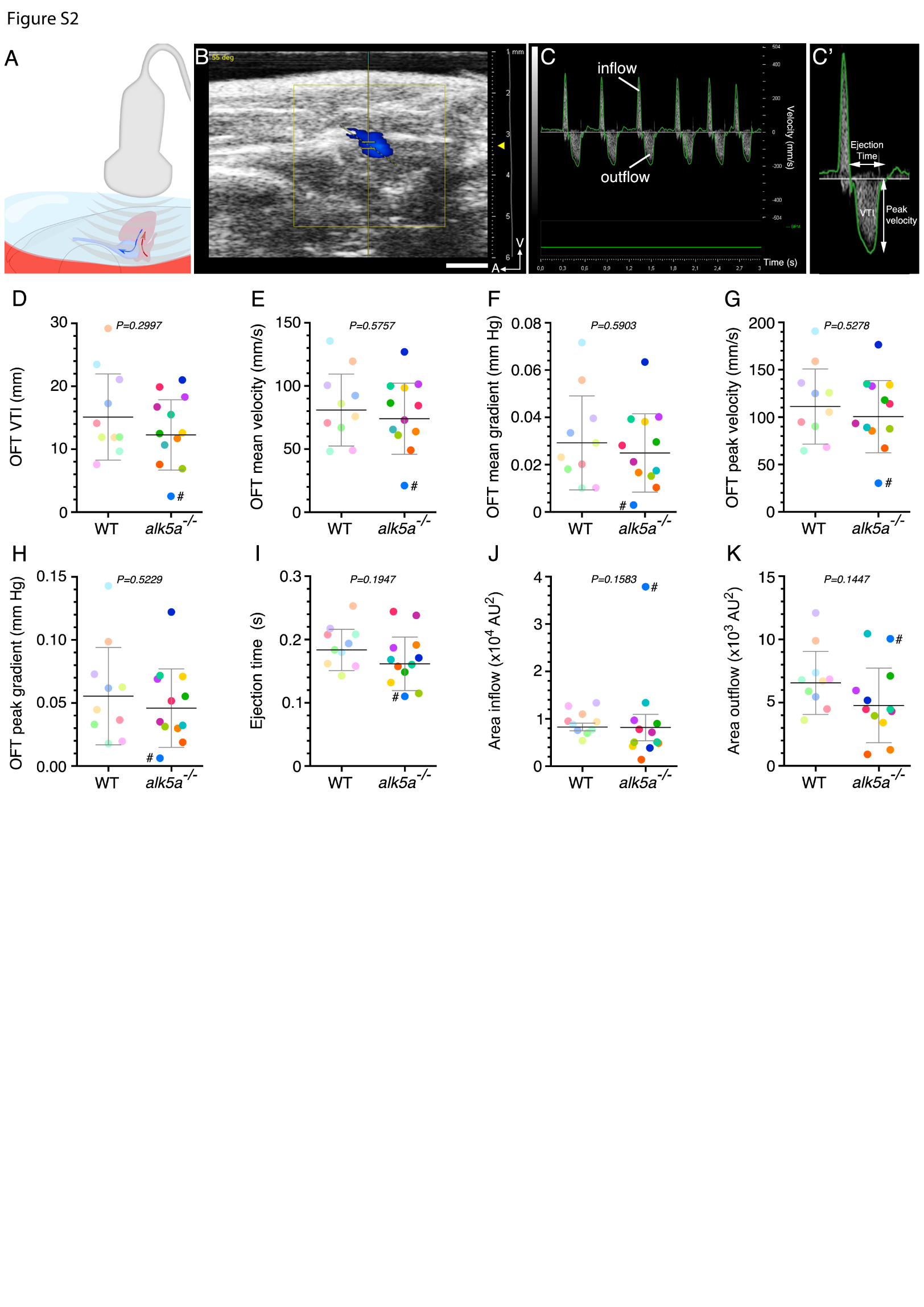
**

**Figure S2 – Most parameters obtained with echocardiography do not show differences between WT** **and *alk5a^-/-^* adult zebrafish.**

A) Schematic of the echocardiography settings, depicting the position of the transducer relative to the heart (red arrow, AVC blood inflow; blue arrow, OFT blood outflow). (B-C’) Quantification of hemodynamic parameters, mostly focused on the OFT flow (blue, B; negative curves, C and C’); VTI, velocity time integral. (D-K) Plotted values for WT (n=10) and *alk5a^-/-^* (n=12) adult zebrafish obtained with Doppler echocardiography analyses. The color of each dot refers to the same zebrafish across all graphs. The dot adjacent to the number symbol (#) identifies the individual zebrafish mentioned in the text. Plots show the values for each individual and the mean ± SD; *P*-values were determined by unpaired *t-*test (D-I, K) or Mann-Whitney test (J). AU – arbitrary units. Scale bar: 1 mm (B).


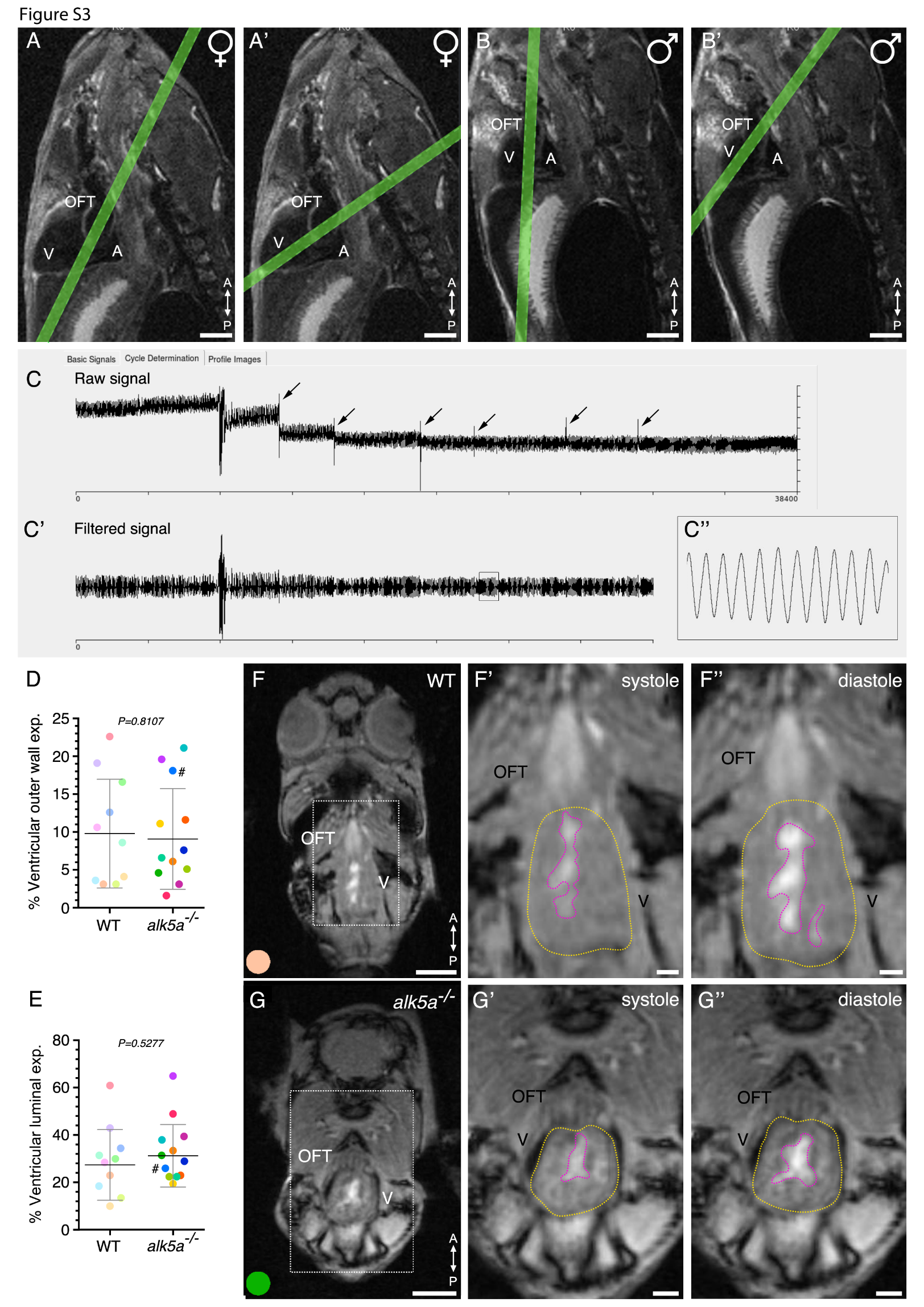


**Figure S3 – MRI analyses of beating hearts do not show significant differences in ventricular expansion between WT and *alk5a^-/^*^-^ adult zebrafish.**

(A-B’) Examples of RARE (Rapid Acquisition with Relaxation Enhancement) images of a female (A, A’) and male (B, B’) WT adult zebrafish; sagittal views. Green bars define the orientation chosen to image OFT (A, B) or ventricular (A’, B’) expansion. (C-C’’) Graphs showing raw signal including heartbeat and sudden movements of the zebrafish (arrows, C) and filtered signal excluding zebrafish movements (C’) to accurately determine heart rate (C’’). Boxed area is shown in C’’. (D, E) Percentage of ventricular outer wall (D) and luminal (E) expansion in WT (n=10) and *alk5a^-/-^* (n=12) adult zebrafish. Plots show the values for each individual and the mean ± SD; *P*-values were determined by unpaired *t-*test (D) or Mann-Whitney test (E). (F-G’’) Single frames of MRI cines of WT (F) and *alk5a^-/-^* (G) adult zebrafish in coronal view. Boxed area is shown in F’, G’ (systole) and F’’, G’’ (diastole). Magenta dashed line, ventricular lumen; yellow dashed line, ventricular outer wall. The color of each dot refers to the same zebrafish across all graphs and images. The dot adjacent to the number symbol (#) identifies the individual zebrafish mentioned in the text. A- atrium, V- ventricle, OFT- outflow tract. Scale bars: 1 mm (A-B’), 1.5 mm (F, G), 500 μm (F’, F’’, G’, G’’).


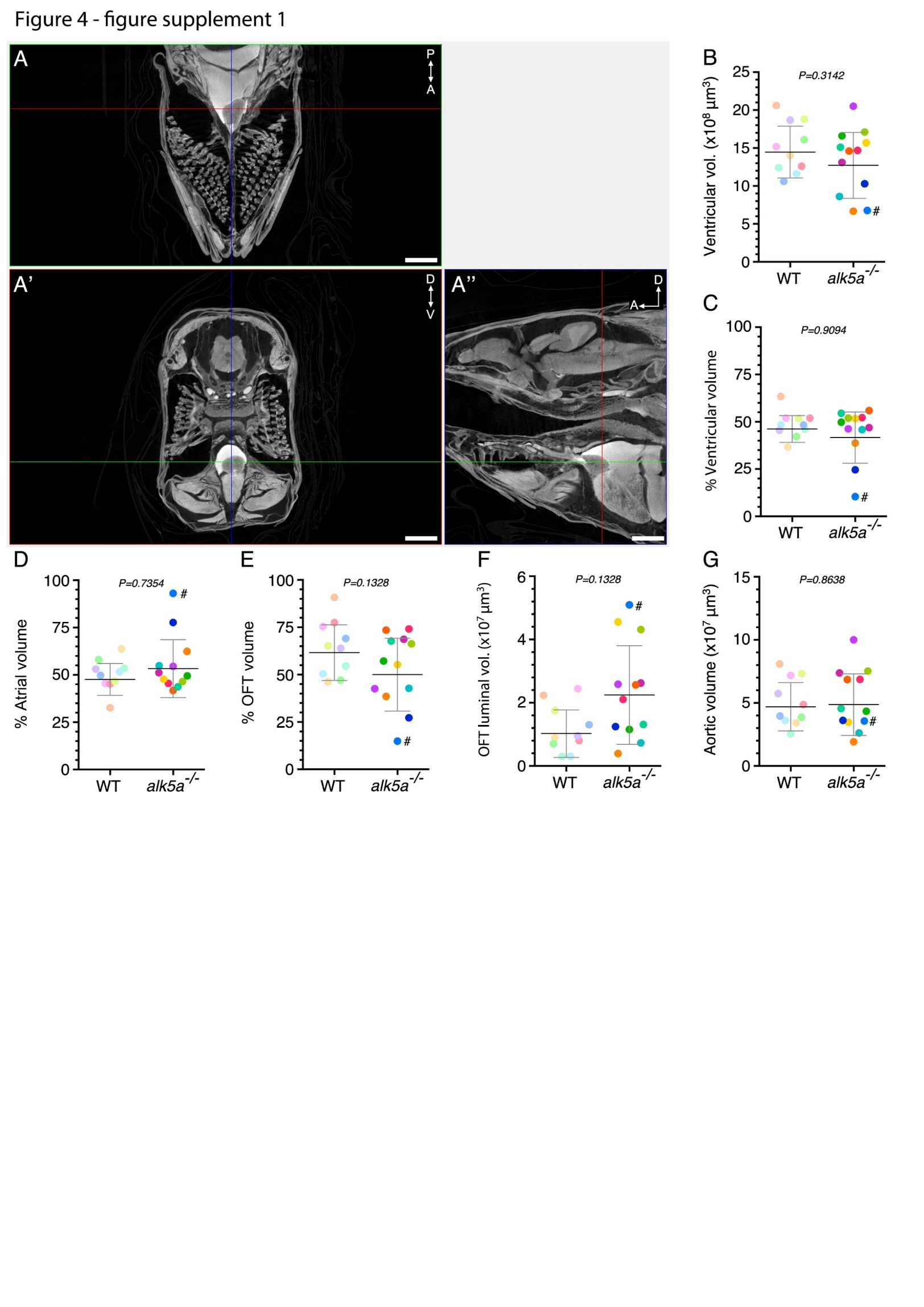


**Figure S4 – All cardiac compartments display a variable volume in *alk5a^-/-^* adult zebrafish.**

(A-A’’) Orthogonal views of a WT adult zebrafish imaged with μ-CT showing coronal (A), axial (A’) and sagittal (A’’) views. (B-G) Quantification of morphological parameters for each cardiac compartment in WT (n=10) and *alk5a^-/-^* (n=12) adult zebrafish. Plots show the values for each individual and the mean ± SD; *P*-values were determined by unpaired *t-*test (B, E-G) or Mann-Whitney test (C, D). The color of each dot refers to the same zebrafish across all graphs. The dot adjacent to the number symbol (#) identifies the individual zebrafish mentioned in the text. Scale bars: 1 mm (A-A’’).

**
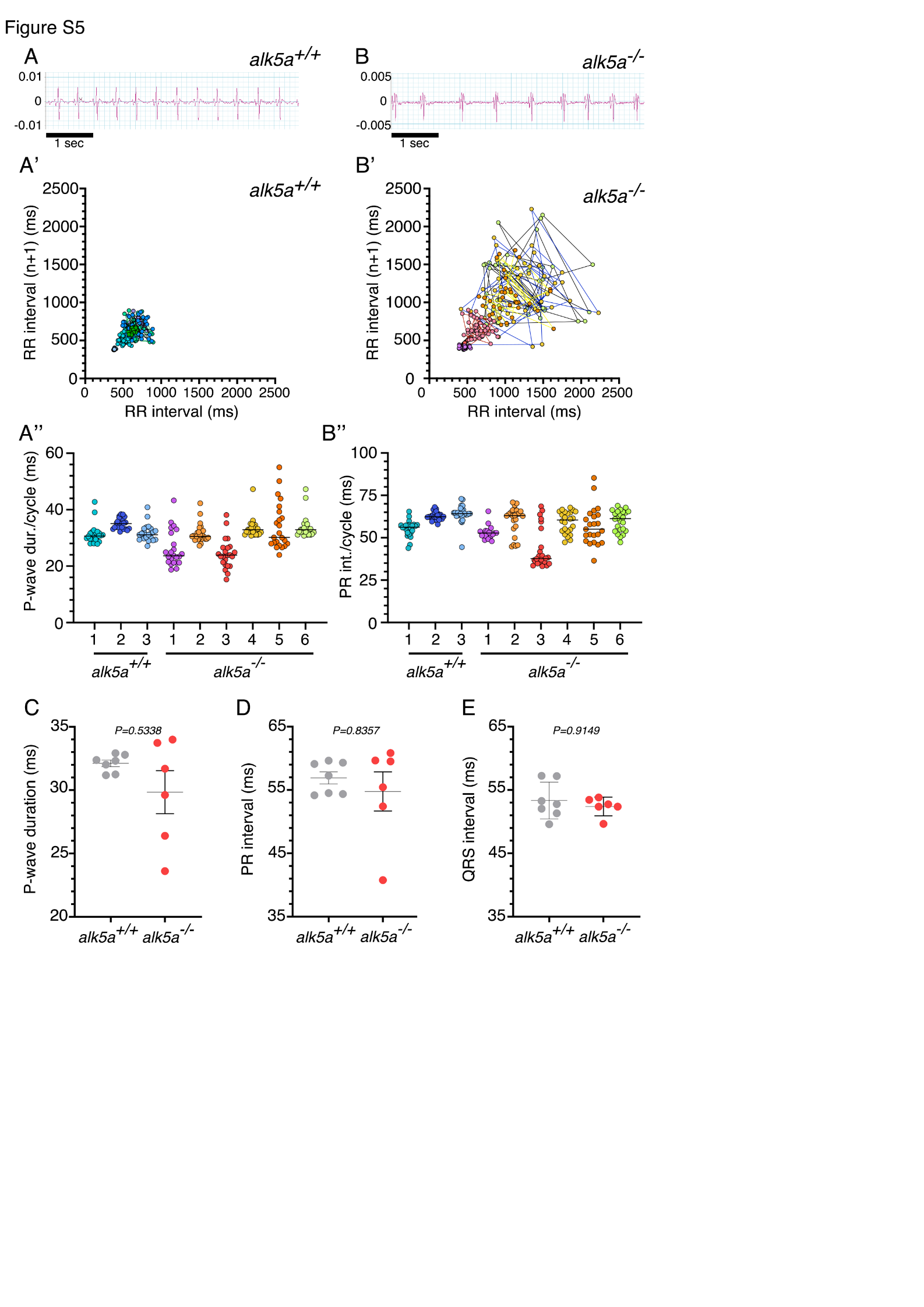
**

**Figure S5 – Electrocardiography analysis shows cardiac arrhythmia in *alk5a^-/-^* adult zebrafish.**

(A-B’) Representative ECG traces (A, B) and Poincaré plots (A’, B’) of consecutive RR intervals in *alk5a^+/+^* (n=7) and *alk5a^-/-^* (n=6) siblings show cardiac arrhythmia in *alk5a^-/-^* zebrafish. P-wave duration and PR interval in each cycle for 3 representative *alk5a^+/+^* zebrafish and all 6 *alk5a^-/-^* zebrafish (A’’, B’’). The color of each dot in A’ and B’ refers to the same zebrafish in graphs A’’ and B’’. Quantification of P-wave duration (C), PR interval (D), and QRS interval (E). Plots show the values for each individual and the mean ± SD; *P*-values were determined by Mann-Whitney test.

**Video Legends**

**Video 1 -** Doppler Echocardiography of a WT zebrafish exhibiting separate blood inflow (red) and outflow (blue), without signs of regurgitation. Related to Figure 2 E, E’.

**Video 2 -** Doppler Echocardiography of an *alk5a*^-/-^ zebrafish exhibiting blood flow regurgitation in the AVC as well as aortic flow directly rostral to the heart. Related to Figure 2 F, F’.

**Video 3 -** Doppler Echocardiography of an *alk5a*^-/-^ zebrafish exhibiting severe blood flow regurgitation in the AVC and OFT. Related to Figure 2 G, G’.

**Video 4 -** MRI imaging of a WT zebrafish in coronal view showing ventricular expansion. Related to Figure S3 F-F’’.

**Video 5 -** MRI imaging of an *alk5a^-/-^* zebrafish in coronal view showing ventricular expansion. Related to Figure S3 G-G’’.

**Video 6 -** MRI imaging of a WT zebrafish in coronal view showing OFT expansion. Related to Figure 3 C-C’’.

**Video 7 -** MRI imaging of an *alk5a^-/-^* zebrafish in coronal view showing OFT expansion. Related to Figure 3 D-D’’.

**Video 8 -** µ-CT scans of the anterior region of a WT zebrafish in sagittal view. Related to Figure 4 A.

**Video 9 -** µ-CT scans of the anterior region of an *alk5a^-/-^* zebrafish in sagittal view. Related to Figure 4 B.

**Video 10 -** Volumetric surface rendering of the cardiac compartments (blue, atrium; red, ventricle; grey, OFT; purple, OFT lumen) of a WT zebrafish imaged with µ-CT. Related to Figure 4 C, C’’.

**Video 11 -** Volumetric surface rendering of the cardiac compartments (blue, atrium; red, ventricle; grey, OFT; purple, OFT lumen) of an *alk5a^-/-^* zebrafish imaged with µ-CT. Related to Figure 4 D, D’’.

**Video 12 -** Volumetric surface rendering of the OFT (grey), OFT volume (purple) and aorta (pink) of a WT zebrafish imaged with µ-CT. Related to Figure 4 C’.

**Video 13 -** Volumetric surface rendering of the OFT (grey), OFT volume (purple) and aorta (pink) of an *alk5a^-/-^* zebrafish imaged with µ-CT. Related to Figure 4 D’.

**Video 14 -** Volumetric surface rendering of the OFT (grey), OFT volume (purple) and aorta (pink) of a WT zebrafish imaged with µ-CT, as seen in a transverse section starting from the OFT and proceeding rostrally. Related to Figure 4 C’’’’.

**Video 15 -** Volumetric surface rendering of the OFT (grey), OFT volume (purple) and aorta (pink) of an *alk5a^-/-^* zebrafish imaged with µ-CT, as seen in a transverse section starting from the OFT and proceeding rostrally. Related to Figure 4 D’’’’.

**Video 16** - Doppler echocardiography of a WT zebrafish in the third (t3) measurement, exhibiting separate blood inflow (red) and outflow (blue), without sign of regurgitation. Related to Figure 7.

**Video 17** - Doppler echocardiography at t1 of an *alk5a*^-/-^ zebrafish treated with isoprenaline exhibiting unidirectional flow in the OFT. Related to Figure 7 D (zebrafish # 1).

**Video 18** - Doppler echocardiography at t2 of an *alk5a*^-/-^ zebrafish treated with isoprenaline exhibiting mild blood flow regurgitation in the OFT. Related to Figure 7 E (zebrafish # 1).

**Video 19** - Doppler echocardiography at t3 of an *alk5a*^-/-^ zebrafish treated with isoprenaline exhibiting stronger blood flow regurgitation in the OFT when compared with t2. Related to Figure 7 F (zebrafish # 1).

**Video 20** - Doppler echocardiography at t3 of an *alk5a*^-/-^ zebrafish treated with isoprenaline showing the most pronounced increase in regurgitation fraction in the OFT from t1 to t3. Related to Figure 7 G (zebrafish # 2).
